# Limiting asymmetric hearing improves benefits of bilateral hearing in children using cochlear implants

**DOI:** 10.1038/s41598-018-31546-8

**Published:** 2018-09-04

**Authors:** Melissa Jane Polonenko, Blake Croll Papsin, Karen Ann Gordon

**Affiliations:** 1 Institute of Medical Science, The University of Toronto, Toronto, ON M5S 1A8 Canada; 2 Neurosciences and Mental Health, The Hospital for Sick Children, Toronto, ON M5G 1X8 Canada; 3 Department of Otolaryngology – Head & Neck Surgery, The University of Toronto, Toronto, ON M5G 2N2 Canada; 4 Otolaryngology – Head & Neck Surgery, The Hospital for Sick Children, Toronto, ON M5G 1X8 Canada

## Abstract

Neurodevelopmental changes occur with asymmetric hearing loss, limiting binaural/spatial hearing and putting children at risk for social and educational challenges. These deficits may be mitigated by providing bilateral hearing in children through auditory prostheses. Effects on speech perception and spatial hearing were measured in a large cohort of >450 children who were deaf and used bilateral cochlear implants or bimodal devices (one cochlear implant and a contralateral hearing aid). Results revealed an advantage of bilateral over unilateral device use but this advantage decreased as hearing in the two ears became increasingly asymmetric. Delayed implantation of an ear with severe to profound deafness allowed asymmetric hearing, creating aural preference for the better hearing ear. These findings indicate that bilateral input with the most appropriate device for each ear should be provided early and without delay during development.

## Introduction

Cochlear implantation has become standard treatment for childhood deafness. One cochlear implant promotes significant gains in speech understanding^1,2^ and language development^3–6^ when provided early in development. On the other hand, access to sound in only one ear results in impaired binaural hearing^7,8^ which is the foundation for sound localization. Without access to spatial hearing, children with asymmetric hearing are at risk for social and educational deficits^7,9,10^. We thus sought to promote bilateral hearing development by providing the most appropriate device in each ear to our large cohort of children with deafness^7^. A cochlear implant was provided in ears with severe/profound deafness; children with bilateral deafness received two cochlear implants whereas children with better hearing in one ear received one cochlear implant and a hearing aid in the other, better hearing, ear (bimodal hearing)^7,11–13^.

Limited or distorted binaural hearing challenges development. Children spend much of their time interacting and learning in dynamic environments, such as the playground and classroom^14–16^ in which they listen to sounds coming from multiple directions. Binaural hearing supports detection of these sounds and the ability to distinguish one sound from another by differences in their spatial locations. To do this, the auditory system largely depends on comparing the level and timing of sounds arriving at the two ears^17^. Even if this binaural coding is impaired^18^, children can take advantage of hearing with both ears by taking advantage of the ear with the better signal-to-noise ratio. This ear has best access to the sound of interest relative to other sounds. The ear closest to the sound source is often relied upon because intensity at the further ear is attenuated by the head (head shadow). Intact binaural hearing further improves listening compared to only listening with the ear that has the better signal-to-noise ratio (binaural squelch) and provides better audibility by combining the signals from each ear (binaural summation)^19^. Restoring children’s access to spatial/binaural cues is thus important to aid hearing in most common situations. Spatial/binaural hearing is clinically evaluated by measuring speech recognition in difficult noise conditions (speech-in-noise) or while listening with both ears over each ear alone (binaural benefit), and measuring the ability to better detect or understand speech when noise comes from different directions than the target speech (spatial unmasking). Asymmetric hearing may distort binaural cues, rendering poorer binaural benefit for speech perception and skewed abilities to detect speech in the presence of other sounds/noise.

The timing of bilateral cochlear implantation is important. As revealed by electrophysiological and functional imaging studies, delaying access to sound in early childhood allows cortical cross-modal plasticity to reorganize auditory areas^20–25^ as well as cortical areas involved in spatial attention and awareness^20,22,26^ but treating only one of two ears with hearing loss leaves the second ear deprived of sound. This creates a new problem termed the “aural preference syndrome”^7^. When hearing is asymmetric, the developing brain reorganizes to preferentially respond to the better hearing ear^21,23,24,27–31^, compromising the ability to process bilateral input^32^. This reorganization occurs within 2–3 years of unilateral hearing and persists even if bilateral input through bilateral cochlear implants or bimodal devices is provided thereafter and used for several years^21,28^. Recent studies suggest that the aural preference syndrome can be reversed if symmetric/balanced bilateral input is provided during early developmental periods^27,28^.

In the present study, we examined whether this neurophysiological support for limited delays to cochlear implantation in children is consistent with functional (behavioural) outcomes. Several groups have reported spatial hearing from small cohorts (<20) of children who received bilateral cochlear implants sequentially after fairly limited (<2 years) bilateral auditory experience^33–39^. These studies revealed better speech thresholds in noise while using two implants, but asymmetric preference for a better signal at the first implanted ear in several children who underwent sequential implantation. Studies with larger cohorts of children (≥50) suggest that children benefit from bilateral implants despite delays to bilateral input but longer delays impair performance in the second hearing ear, creating asymmetric abilities between the two ears for understanding speech^1,40–43^ and for spatial hearing^44,45^. One of the main problems of relating existing speech perception data to electrophysiological findings is that only a few of the behavioural studies include children with very short or no delays to bilateral implantation^1,45^, leaving a question about the most appropriate timing of bilateral input to prevent behavioural consequences of aural preference.

Behavioural data from children using bimodal devices are also needed. Whereas electrophysiological data included children with a range of hearing in the non-implanted ear (normal to severe/profound)^13,27,28^, previous studies largely focused on children who have significant (severe/profound) hearing loss in their non-implanted ear^46–50^. These studies reveal benefits of bimodal hearing over the use of a cochlear implant alone but continued challenges for listening to speech in noise. Data from some children with better hearing in the non-implanted ear also reveal bimodal improvements in speech perception and spatial hearing that depend on duration of deafness in the poorer ear and access to consistent sound in the better ear^11,12,28,51^. The cohorts of bimodal users represent a very diverse population of implant users^28,51–54^. Asymmetric hearing loss in children appears to have an increased incidence of auditory nerve hypoplasia, enlarged vestibular aqueducts and positive cytomegalovirus^28,52,54^. These etiologies are associated with acquired and/or progressive hearing loss which could mitigate some of the deleterious effects of inter-implant delay^35,40,42^. Accordingly, the present study aimed to characterize the pre-implantation hearing histories of all children and to use this information to predict lasting effects of the degree and duration of asymmetric hearing on speech perception and spatial hearing.

Given the importance of bilateral input during development, we asked whether providing bilateral input through bilateral cochlear implants or bimodal devices worked to promote symmetric functional outcomes in a large diverse cohort of children, thereby preventing functional aural preference. We hypothesized that the benefit of bilateral input: (1) increases with earlier access to bilateral input; (2) does not need to be restricted to one mode of stimulation/hearing and (3) is related to hearing experience/demographic information. Results suggest that listening through bilateral devices is better than with one device, and the best timing for intervention is to provide bilateral devices as early as possible. Decisions regarding the type of device should consider the degree of residual hearing and asymmetry between ears.

## Results

### Principle component analysis of hearing history differentiates groups of children receiving bilateral devices

Group demographic details are described in Table 1. To better understand the largest sources of variation in several related demographic variables of the hearing histories in children with bilateral devices (ANOVA group comparisons for each variable, *p* < 0.0001; Table 1), a principal component (PC) analysis was completed after log-transforming and standardizing the following pre-implantation variables: age at first implantation; unaided pure-tone-average of 0.5, 1, 2 kHz (PTA) in the first and second implanted ears or ear that maintained HA use; pre-implantation unaided PTA asymmetry; post-implantation (bilateral devices) aided PTA asymmetry; and durations of asymmetric hearing loss, unilateral deafness (thresholds ≥ 90 dB HL), bilateral deafness and pre-implantation acoustic hearing. Mean ± SD for these variables by group are summarized in Table 1B. Complete data for all these variables, available in 361/461 (78.3%) children, were used in the PCA. Three components with eigenvalues >1 were extracted, which together explained 69% of the variance in hearing histories. By combining several related demographic variables into components, the comprehensive hearing history could be considered when predicting behavioural outcomes.

**Table 1:** Group numbers and mean ± SD of time- and hearing-based factors used in the principal component analysis, and of the first 3 principal components.

Variable		Bimodal Devices (HA + CI) (*n* = 80)	Bimodal Sequential BiCI (*n* = 18)	Sequential BiCI (*n* = 170)	Simultaneous BiCI (*n* = 154)	Older Simultaneous BiCI (*n* = 39)	Statistic *p < 0.05
**A. Factors not in PCA**							
Implanted ear: Left/Right (% Right)		40/40 (50.0%)	7/11 (61.1%)	33/137 (80.6%)	n/a	n/a	*Χ*^2^(2) = 24.8*
Gender: Female/Male (% Male)		31/49 (61.3%)	5/13 (72.2%)	79/91 (53.5%)	59/95 (61.7%)	22/17 (43.6%)	*Χ*^2^(4) = 7.3
Hearing loss: Symmetric/Asymmetric (% Asymmetric)		20/60 (75.0%)	8/10 (55.6%)	142/11 (7.2%)	139/6 (4.1%)	24/11 (34.1%)	*Χ*^2^(4) = 201.3*
Bilateral device use (years)	Speech perception test	3.2 ± 2.9	2.0 ± 1.4	3.8 ± 2.8	5.1 ± 2.2	2.5 ± 1.8	*F*(4,432) = 14.1*
	Spatial unmasking test	3.2 ± 3.0	2.4 ± 1.5	6.5 ± 3.1	5.1 ± 2.7	2.8 ± 1.9	*F*(4,164) = 10.3*
**B. Factors in PCA**							
Age (years)	Age at CI1	7.2 ± 4.6	6.7 ± 3.9	3.3 ± 3.3	1.7 ± 1.0	10.0 ± 4.4	*F*(4,455) = 78.4*
Duration of experience (years)	Pre-CI acoustic hearing	6.0 ± 4.6	5.2 ± 3.9	0.6 ± 1.5	0.2 ± 0.6	5.7 ± 4.6	*F*(4,413) = 91.6*
	Asymmetric hearing	2.4 ± 3.4	1.6 ± 3.1	0.1 ± 0.7	0.0 ± 0.2	0.4 ± 1.8	*F*(4,411) = 28.5*
Duration of deafness (years)	Unilateral	1.6 ± 2.7	1.1 ± 2.9	0.1 ± 0.7	0.0 ± 0.2	0.4 ± 1.8	*F*(4,411) = 17.9*
	Bilateral	0.1 ± 0.6	0.3 ± 0.6	1.1 ± 1.8	0.8 ± 0.5	1.6 ± 2.8	*F*(4,413) = 10.2*
Pre-CI unaided PTA (dB HL)	CI1 ear	93.4 ± 18.2	94.2 ± 19.5	107.5 ± 13.8	104.8 ± 14.0	95.1 ± 13.6	*F*(4,400) = 15.3*
	CI2/HA ear	67.5 ± 22.7	84.0 ± 7.5	107.0 ± 14.6	104.6 ± 14.2	99.4 ± 12.1	*F*(4,420) = 94.3*
Hearing asymmetry (dB)	Pre-CI unaided	27.9 ± 26.9	17.9 ± 15.7	6.9 ± 7.5	6.6 ± 7.4	10.0 ± 11.8	*F*(4,399) = 35.1*
	Post-CI aided	10.0 ± 7.2	5.6 ± 5.8	4.0 ± 4.1	3.0 ± 3.1	1.9 ± 1.7	*F*(4,393) = 31.8*
**C. Main PCA Components (Eigenvalues > 1)**							
PC1: Asymmetry		−4.6 ±2.2	−3.2 ±1.8	0.3 ± 1.3	0.7 ± 1.1	−1.0 ±1.6	*F*(4,356) = 168.1*
PC2: Unilateral deafness		−2.2 ±2.9	−1.0 ±2.8	−0.9 ±1.4	−0.7 ±1.1	0.9 ± 1.2	*F*(4,356) = 19.67*
PC3: Any deafness (unilateral and/or bilateral)		5.7 ± 4.9	2.7 ± 4.0	1.8 ± 2.8	1.3 ± 2.1	−0.3 ±1.5	*F*(4,356) = 28.22*

CI = cochlear implant; HA = hearing aid; BiCI = bilateral cochlear implants; PTA = pure-tone average of 0.5, 1, 2 kHz hearing thresholds; PC = principal component; CI1 = first implanted ear; CI2 = second implanted ear.

Note: Groups were compared using a Chi-Square test for proportion variables and ANOVA for continuous variables. The corresponding Chi-squared and F statistics are provided in the last column.

**Figure 1:**
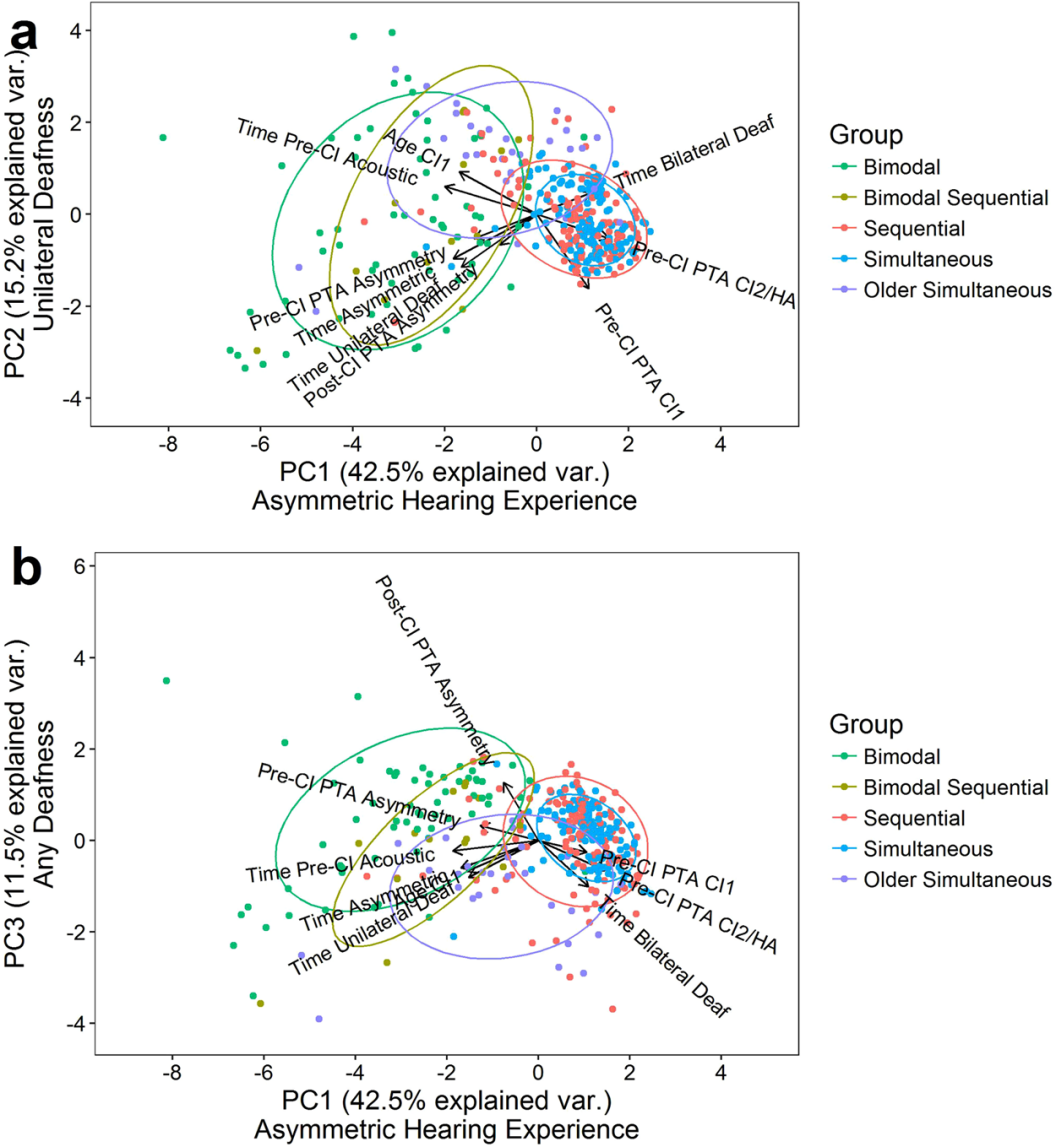
Principal component analysis of hearing histories. Principal component (PC) scores and their loading vectors are displayed for (**a**) PC2 and (**b**) PC3 relative to PC1, which together explained 69% of the variance in the hearing histories of each group of bilateral device users. Variables contributing highly to PC1 related to the asymmetric hearing experience whereas the combination of highly contributing variables to PC2 and PC3 correspond to unilateral deafness or any type of deafness (unilateral and bilateral) respectively. Ellipses represent 68% (±1 SD distributions) of the scores for each group, which are differentiated by colour.

To describe what each PC encompassed, variables which contributed proportionally more to the PC than expected from equal contributions were considered (i.e., >100%/9 variables = 11.1%). The first two components together explained 57.7% of the variance in hearing history and both included variables associated with asymmetric hearing experience prior to implantation (age at CI1, duration of asymmetric hearing, and duration of unilateral deafness). However, additional variables that differed between the two components contributed to different aspects of the asymmetric hearing experience. The first component (PC1) individually explained 42.6% of the variance in hearing history and, along with the 3 variables that were related to pre-implant asymmetric hearing experience, included variables associated with the duration and degree of residual acoustic hearing (duration of pre-acoustic hearing and unaided PTA in the CI2/HA ear). This first component thus reflected the contribution of the better ear to the asymmetric hearing experience. The second component included the unaided PTA in the CI1 ear, reflecting the contribution of residual hearing or deafness in the poorer hearing ear to the asymmetric hearing experience. This second component explained 15.1% of the variance. A third component, explaining 11.4% of the variance, was associated with any deafness or poor residual hearing pre- or post-implantation (duration of unilateral and bilateral deafness, unaided PTA in CI2/HA ear, post-CI asymmetry in aided PTA). The relationships between components are plotted in Fig. 1 (a shows PC1 and PC2 and b shows PC1 and PC3). Also shown by arrows are the correlation coefficients (factor loadings) of the variables with each component. The factor loading matrix of the PCA is provided in Supplemental Table 1; shaded and bolded factors most contributed to each PC.

Groups were clearly identified by differences in PC scores (Table 1C, ANOVA *p* < 0.001). PC1 differentiated groups with varying degrees and durations of asymmetry: bimodal users experienced the most asymmetry (negative PC1 values) followed by bimodal sequential users (Tukey HSD post-hoc test *p* = 0.008). The older simultaneous group had significantly less asymmetric hearing than both bimodal groups (both *p* < 0.001) but significantly more than the bilateral simultaneous (*p* < 0.001) and sequential (*p* < 0.001) groups, who both experienced very minimal asymmetry (*p* = 0.17). This is consistent with the number of children categorized as having (a)symmetric hearing loss and the mean PTA asymmetries in Table 1A,B. PC2 differentiated groups with residual or unilateral hearing: older simultaneous users experienced periods of progressive bilateral hearing loss (higher PC2 scores, all *p* < 0.001) compared to the minimal hearing experience of sequential and simultaneous bilateral CI users prior to implantation (PC2 scores around zero). This contrasts with the negative PC2 scores of bimodal groups (all *p* < 0.01) who had better residual hearing in the CI1 ear (*p* < 0.001), later implantation (*p* < 0.001), and longer durations of asymmetric hearing (*p* < 0.001) and unilateral deafness (*p* < 0.001). Although bimodal and bimodal sequential groups significantly differed by asymmetric (PC1 *p* = 0.008) but not deafness-related PC scores (PC2 *p* = 0.08; PC3 *p* = 0.11), bimodal sequential users had worse residual hearing in the ear that kept the HA while waiting to receive a second implant (*p* = 0.001), and consequently tended to have less hearing asymmetry pre-CI2 (*p* = 0.069).

### Most children rapidly achieve open-set speech perception skills after bilateral/bimodal device use

**Figure 2:**
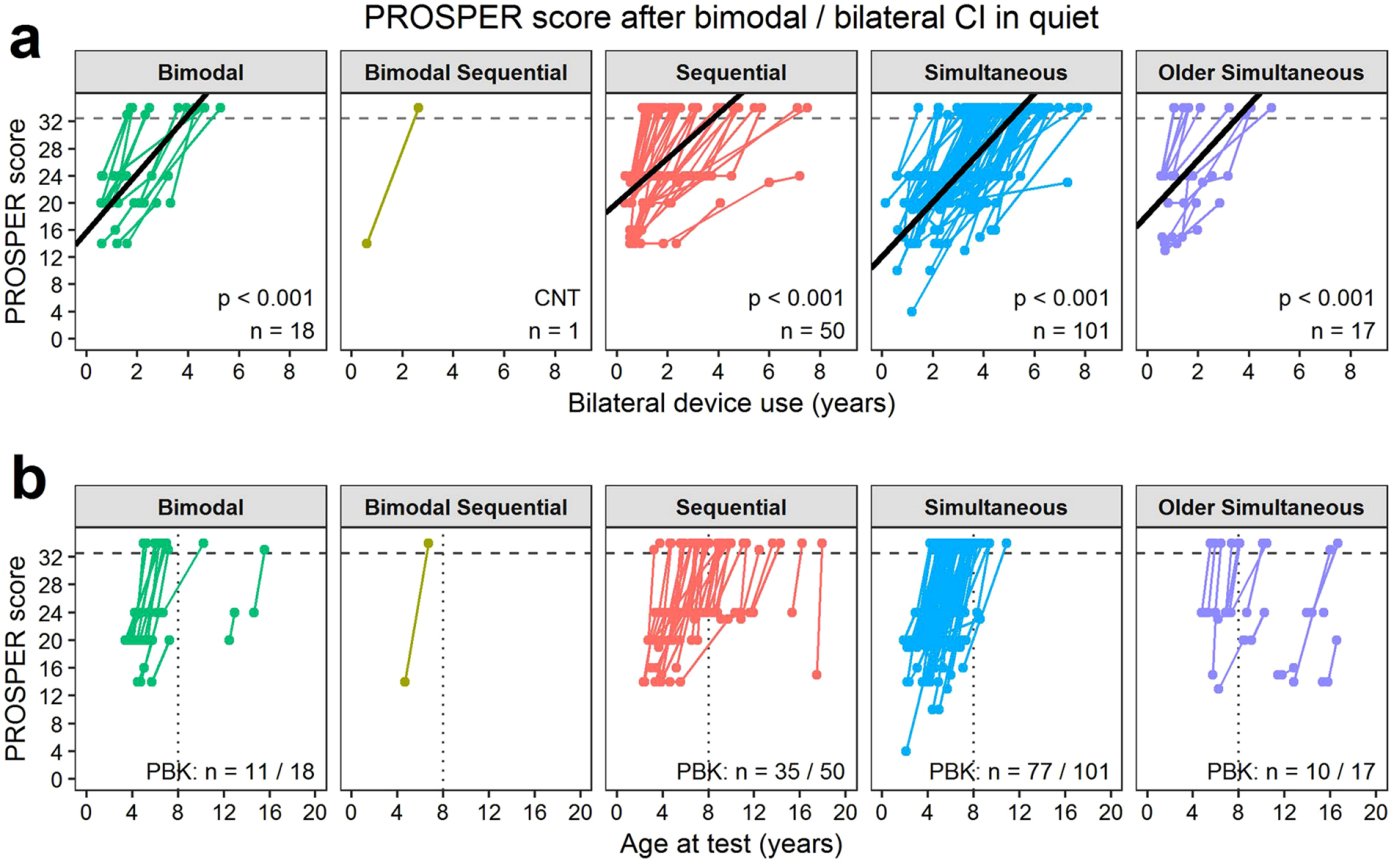
Progressive speech perception testing with time. (**a**) The Pediatric Ranked Order Speech Perception (PROSPER) score for the CI1-quiet condition increased with bilateral device experience (linear mixed-effects regression with likelihood ratio test: *χ*^2^(1) = 219.4, *p* < 0.001; solid black lines) at a similar rate for each group (*χ*^2^(3) = 3.9, *p* = 0.274). This measure hierarchizes accuracy (<50%, ≥50%) and test from simple detection to open-set word recognition. The dashed gray line delineates scores for the most challenging PBK words test (≥33). Coloured lines join scores for each child across time. The bottom right corner of each panel displays the number of children (*n*) in each group for whom repeated testing was available with initial scores < 33. (**b**) Most children progressed to testing with the PBK (numbers indicated at the bottom right of each panel) but this occurred after age 8 years (dotted line) in more of the sequential and older simultaneous bilateral implant users than bimodal and simultaneous users.

The first analyses examined longitudinal changes in speech perception accuracy on standardized tests after activating bilateral input through bilateral CIs or bimodal hearing. The same test was administered to each ear in quiet and noise during a single session, so the Pediatric Ranked Order Speech Perception (PROSPER) score was similar for both ears and condition at each test time. Representative data for CI1 in quiet are plotted Fig. 2. Of 307 children tested repeatedly, 186 (60.6%) children with initial scores < 33 (i.e., easier tasks than the PBK) progressively improved by 4.3 ± 0.6 PROSPER scores per year of bilateral device use (linear mixed effects regression; likelihood ratio test *χ*^2^(1) = 219.4, *p* < 0.001; Fig. 2a). This means that children advanced through two tests each year. The rate of change with bilateral device experience was similar for all groups (*χ*^2^(3) = 3.9, *p* = 0.274) but the intercept varied by group (*χ*^2^(3) = 63.9, *p* < 0.001). Simultaneous users began their bilateral hearing with easier tasks (−4.0 ±1.2 PROSPER score; 2 tests) and sequential users started with a harder task (2.7 ± 1.3 PROSPER score; 1 test) than the bimodal and older simultaneous groups, which partly reflected their older age at bilateral device use (Fig. 2b). This meant that, of the children who reached the milestone of testing by PBK (133/186; 71.5%), 82% of bimodal users (9/11) and 84% of the simultaneous bilateral CI group (65/77) were under 8 years of age, whereas only approximately half of the sequential (17/35; 48.6%) and older simultaneous (5/10; 50.0%) bilateral CI groups could be tested with the PBK by 8 years of age (Fisher’s Test *p* < 0.001; Fig. 2b).

### Asymmetric speech perception develops in some children

**Figure 3:**
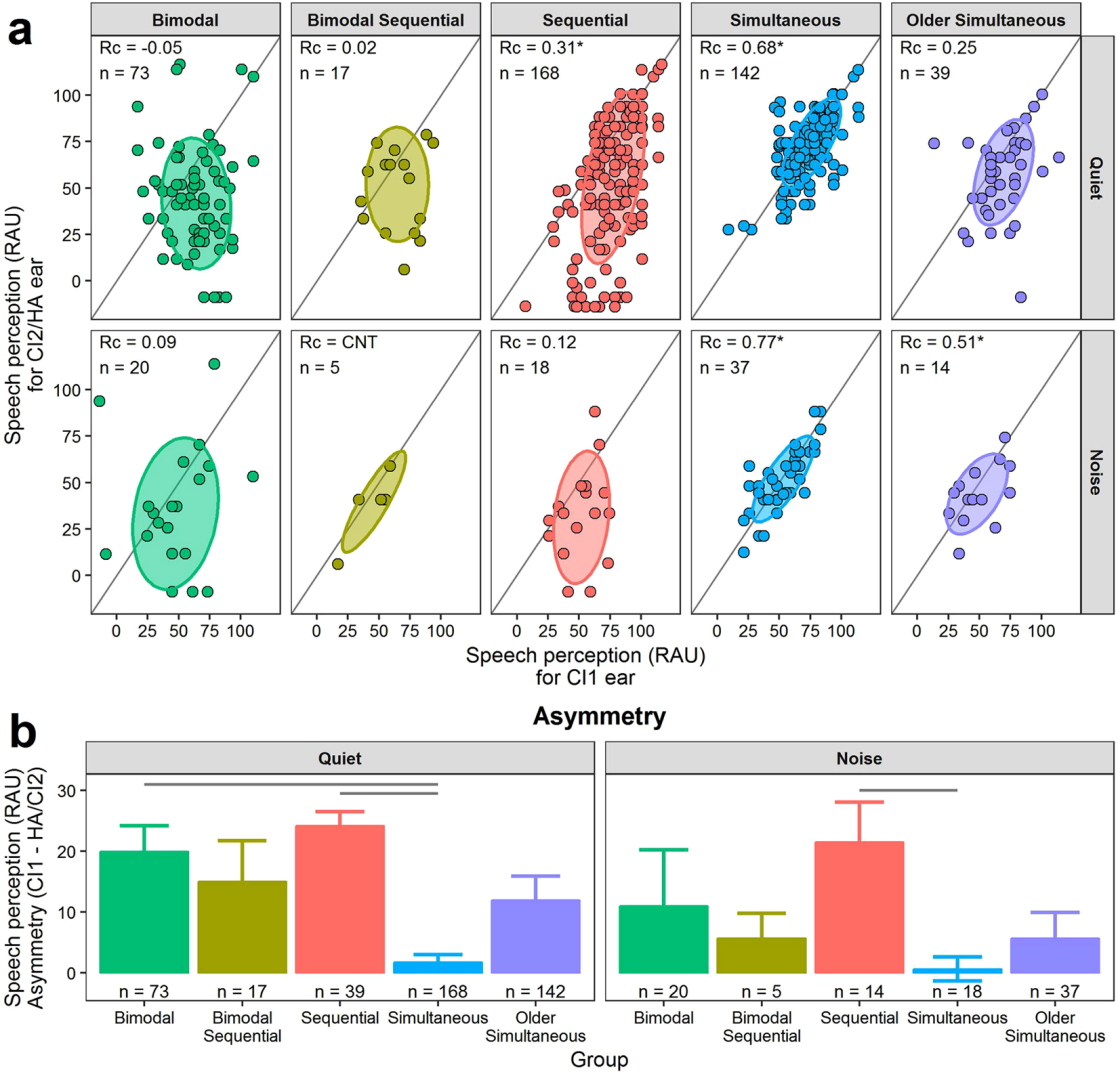
Asymmetry in speech perception between each ear tested in quiet and noise. (**a**) Speech perception in rationalized arcsine units (RAU) of the ear with the hearing aid (HA) or second cochlear implant (CI2) was worse than that of the first implanted ear (CI1) for all but the simultaneous groups when measured in quiet or +10 dB SNR noise, as indicated by ±1 SD (68% of data) ellipses residing below the gray unity lines. Concordance correlation coefficients (*Rc*) and number of children (*n*) are provided for each group and condition; asterisks indicate when the 95% confidence interval of the *Rc* estimate does not cross zero. (**b**) Mean (±SE) asymmetry in speech perception (RAU) was greatest in bimodal and sequential bilateral CI users and smallest in young simultaneous bilateral CI users. Positive values indicate better scores for CI1. Gray lines denote post-hoc Tukey HSD comparisons with *p* < 0.05. CNT = could not test (too few children); CI = cochlear implant; HA = hearing aid.

Functional aural preference was measured as asymmetric speech perception between the two ears using scores from the latest available test. Most were PBK test scores (333/439, 75.8%). The simultaneous group used bilateral devices the longest (*p* < 0.001) but all groups used both devices for at least 2 years (Table 1A). Asymmetry in speech perception is shown in Fig. 3. In Fig. 3a, speech perception scores for the ear with the HA (bimodal users) or CI2 (bilateral CI users) are plotted against those of the CI1 ear. In quiet, at least 68% (±1 SD) of children in all but the simultaneous group had better speech perception scores using CI1 than CI2/HA, as indicated by ellipses and data points below the unity line, and small (< ± 0.4) concordance correlation coefficients. The same asymmetries were found for sequential and bimodal groups when testing in noise, although overall scores were poorer (Fig. 3a), whereas speech perception continued to be symmetric between the ears in both simultaneous groups. Only a few bimodal sequential bilateral CI users were tested in noise.

Mean ± SE asymmetry (Fig. 3b) favoured CI1 across groups (insufficient bimodal sequential data in noise for analyses) but the degree of asymmetry varied by group (ANOVA, quiet: *F*(4,434) = 14.6, *p* < 0.001; noise: *F*(3,85) = 2.9, *p* = 0.038). Speech perception was more asymmetric for bimodal and sequential groups than the simultaneous group in quiet (both Tukey HSD post-hoc *p* < 0.001), and for sequential compared to simultaneous bilateral CI users in noise (Tukey HSD post-hoc *p* = 0.032).

Longitudinal measures in both ears were available in 307 (69.9%) children and revealed little change in asymmetry using linear mixed effects regression (0.6 ± 0.3 RAU/year of age; *χ*^2^(1) = 5.4, *p* = 0.02; see Supplemental Fig. 1). There was no significant change in slope by group (interaction: *χ*^2^(4) = 5.6, *p* = 0.20) but, consistent with Fig. 3, the degree of asymmetry differed by group (intercept: *χ*^2^(4) = 66.8, *p* < 0.001): bimodal and sequential groups had the greatest asymmetry (25.7 and 22.0 RAU respectively) and simultaneous groups had the least (−1.1–1.3 RAU).

### Bilateral advantage in quiet and noise for all groups

**Figure 4:**
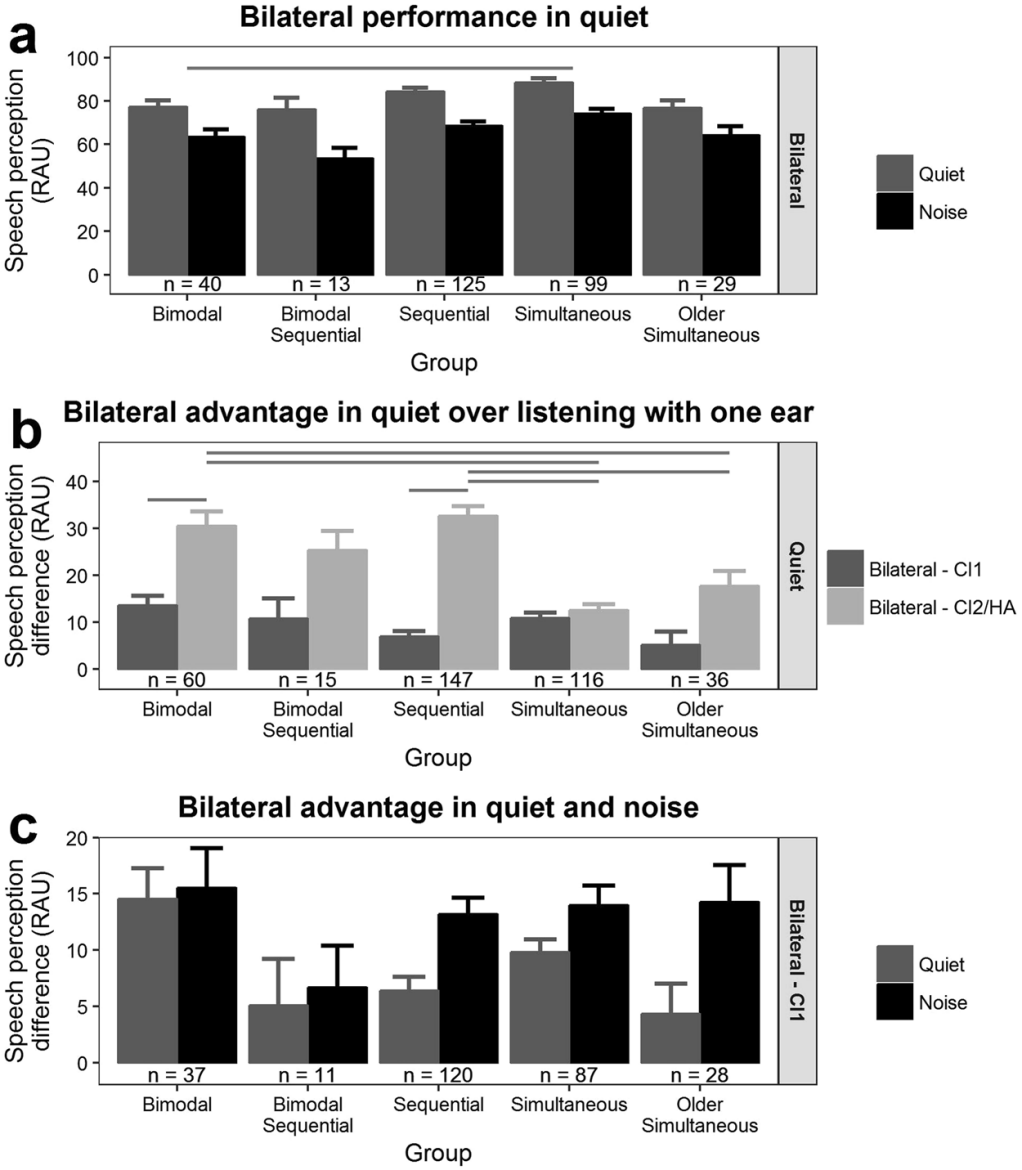
Bilateral advantage for speech perception. (**a**) Mean ± SE bilateral speech perception accuracy was greater in quiet than in noise for all groups. Simultaneous bilateral cochlear (CI) implant users were more accurate than bimodal users (post-hoc Tukey HSD comparison *p* < 0.05). (**b**) Bilateral advantage was calculated as: bilateral - unilateral speech perception. Mean ± SE bilateral advantage is provided. In quiet, all groups experienced bilateral benefit over listening with only one ear. There was a group x ear interaction in bilateral advantage. The bilateral advantage to speech perception was greater compared with HA/CI2 alone versus CI1 alone for bimodal and sequential users. The bilateral advantage over HA/CI2 was greater for bimodal and sequential users than both simultaneous and older simultaneous groups. Gray lines indicate significant post-hoc Tukey HSD comparisons (*p* < 0.05). (**c**) The bilateral advantage over listening with only CI1 was greater in noise than quiet across groups (main effect of condition only).

Mean ± SE bilateral speech perception scores in quiet and in noise are presented in Fig. 4a. For each group, mean accuracy was ≥75 RAU in quiet but < 75 RAU in noise. While accounting for the significant effect of stimulus delivery method (see Supplemental Fig. 6) in a repeated measures ANOVA, bilateral scores differed by group (*F*(4,299) = 5.4, *p* < 0.001) and condition (*F*(1,299) = 349.9, *p* < 0.001) but there was no interaction between group and condition (*F*(4,299) = 1.3, *p* = 0.26). Bilateral accuracy was 8.8 ± 2.4 RAU greater for quiet than noise (Tukey HSD post-hoc *z* = 3.6, *p* < 0.001), and simultaneous bilateral CI users were 10.7 ± 3.5 RAU more accurate than bimodal users (*z* = 3.1, *p* = 0.017).

The potential advantage of bilateral CI and bimodal device use relative to each ear was examined. As shown in Fig. 4b, all groups demonstrated a significant advantage of bilateral device use over listening with one ear alone (independent *t*-test *µ* = 0, *p* < 0.05) but the degree of benefit significantly differed by ear and group (repeated measures ANOVA: *F*(4,369) = 14.7, *p* < 0.001). In simultaneous and bimodal sequential groups, the bilateral advantage was equal regardless of which ear was the unilateral reference (Tukey HSD post-hoc *p* > 0.05). Conversely, bilateral benefit was asymmetric with more benefit relative to the overall poorer ear alone for bimodal (the HA: Tukey HSD post-hoc *z* = 5.6, *p* < 0.01) and sequential bilateral CI users (the CI2: Tukey HSD post-hoc *z* = 12.5, *p* < 0.01). Figure 4c shows the mean ± SE bilateral advantage over listening with CI1 (often the stronger ear) alone in both quiet and noise. Irrespective of group (repeated measures ANOVA *F*(4,278) = 1.8, *p* = 0.13), bilateral advantage was 4.2 ± 1.7 RAU greater in noise than in quiet (repeated measures ANOVA *F*(1,278) = 6.3, *p* = 0.013) which highlights the importance of bilateral input in challenging acoustic conditions. Bilateral advantage over CI2/HA in noise is plotted in Supplemental Fig. 2.

### Symmetric speech perception is needed for greatest advantage of bilateral hearing

**Figure 5:**
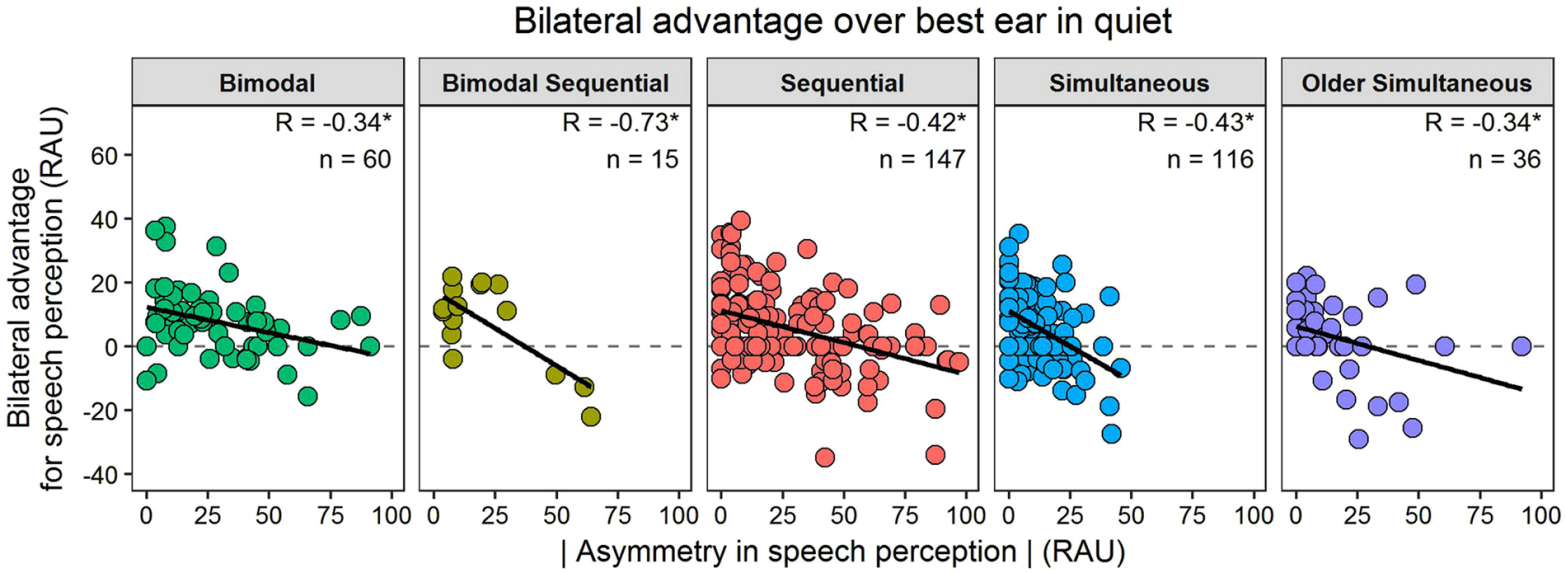
Asymmetry and bilateral advantage for speech perception are related. For each group the advantage of bilateral input over the best performing ear alone decreased as the absolute asymmetry in speech perception in quiet increased. Correlation coefficients (*R*) and number of children (*n*) are provided for each group and condition. Asterisks indicate correlations with *p* < 0.05.

Figure 5 shows the advantage of bilateral input for speech perception over listening with the best performing ear alone. Regression lines indicate a decreasing bilateral benefit as absolute asymmetry between the two ears increases (*R* = −0.34 to −0.73, *p* < 0.05). A similarly negative relationship was found between the advantage of adding bilateral hearing to hearing with CI1 alone and asymmetry in speech perception (Supplemental Fig. 3). There was no relationship between absolute bilateral speech perception accuracy and asymmetry in speech perception, or with principle components or hearing history variables comprising the principle components (*p* > 0.05).

### Children with bilateral/bimodal devices benefit from spatial hearing

**Figure 6:**
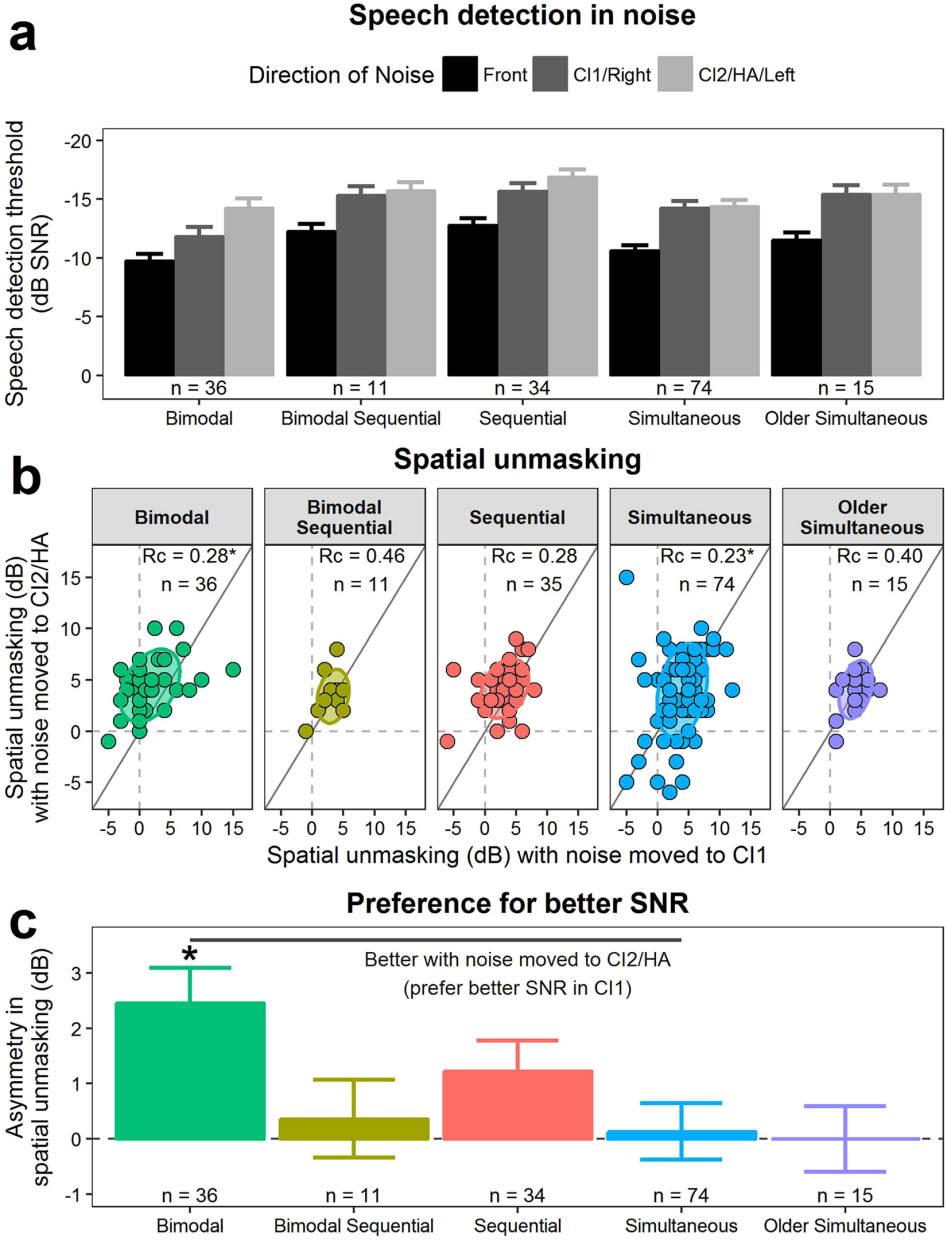
Speech detection and spatial unmasking in noise. (**a**) Mean ± SE speech detection thresholds with speech-weighted noise to the side of the first implanted ear (CI1; dark gray) or to the side of ear with the second cochlear implant (CI2) or hearing aid (HA) (light gray) were better (more negative) than with noise coming from the front (black). Note the reversed scale; better (negative) scores are shown going upwards. (**b**) Most children benefited from spatial unmasking (values > 0) from when noise was moved from the front to the side of CI2/HA versus from the front to the side of CI1. Gray lines denote unity, ellipses represent the ±1 SD (68%) distribution of response in each group. The concordance correlation coefficient (*Rc*) is given for each group, with an asterisk identifying when the 95% confidence interval does not cross zero. (**c**) Mean ± SE difference in spatial unmasking between moving noise from front to either side (asymmetry). The asterisk indicates a significant difference from zero (FDR-corrected *p* < 0.05) based on independent *t*-tests and the gray line denotes significant difference between groups based on ANOVA (*p* < 0.05).

Mean ± SE detection thresholds for speech in noise are presented in Fig. 6a. Age at test differed by group (ANOVA *F*(4,166) = 41.4, *p* < 0.001): simultaneous users were younger than bimodal users (Tukey HSD post-hoc *p* < 0.001), and both of these groups were younger than the other three groups (*p* < 0.05). Furthermore, simultaneous and sequential groups had longer bilateral device use than the other groups (all *p* < 0.05; Table 1A). Because younger children are expected to have worse speech thresholds in noise^33,55,56^, age was added as a covariate to subsequent analyses. Speech detection thresholds were affected by direction of noise (repeated measures ANOVA *F*(2,328) = 9.3, *p* < 0.001) but not group (*F*(4,164) = 2.2, *p* = 0.07) or age (*F*(1,164) = 3.3, *p* = 0.07). Speech detection thresholds improved (more negative) when noise was spatially separated from the speech (spatial unmasking) (Tukey HSD post-hoc *z* < −3, *p* < 0.001) and was best when noise was directed towards CI2/HA (*z* = −2.6, *p* = 0.031).

To further explore these differences, the degree of spatial unmasking (thresholds with collocated - spatially separated speech and noise) was calculated for each noise direction (Fig. 6b). Most children had positive (improved) spatial unmasking values. The ±1 SD distribution ellipses (68% of data) and data points above the unity line for the bimodal and sequential groups suggest these groups tended to derive more spatial unmasking when noise was directed to CI2/HA. Accordingly, concordance correlation coefficients were small (<±0.4) or insignificant. Bimodal sequential and both simultaneous groups had ±1SD distributions along the unity line indicating overall symmetric spatial hearing. Age did not impact spatial unmasking (covariate in repeated measures ANOVA *F*(1,164) = 0.01, *p* = 0.92) but there was a significant interaction between group and direction of noise (*F*(4,164) = 2.6, *p* = 0.035), whereby bimodal users experienced (mean ± SD) 2.5 ± 0.6 dB greater spatial unmasking with noise directed to the HA than CI1 (Tukey HSD post-hoc *z* = 3.9, *p* < 0.01). Overall, the groups experienced 3.3 ± 3.6 dB (bimodal group) to 3.9 ± 2.1 dB (older simultaneous group) spatial unmasking.

Asymmetric spatial unmasking between the two sides could relate to aural preference. As shown in Fig. 6c, asymmetric spatial unmasking differed across groups (ANOVA *F*(4,14) = 2.5, *p* = 0.041) regardless of age (*F*(1,164) = 0.5, *p* = 0.48). Bimodal (2.5 ± 0.6 dB, independent *t*-test *t*(35) = −3.9, FDR-adjusted *p* = 0.002) and sequential bilateral CI users (1.2 ± 0.6 dB, independent *t*-test *t*(33) = −2.2, FDR-adjusted *p* = 0.088) had notable asymmetries. Asymmetry in the bimodal group, in particular, was greater than the test step-size of 2 dB and greater than the asymmetry in simultaneous users (difference: 2.3 ± 0.2 dB; Tukey HSD post-hoc *p* = 0.026). This preference for a better signal-to-noise ratio in CI1 occurred in the sequential users despite similar access to sound in each ear as measured by aided pure-tone average thresholds; the difference between the two CIs in this group was 4.1 ± 4.1 dB which was less than the test step-size of 5 dB (Table 1B). The asymmetric use of spatial separation in bimodal users could partly reflect the 10.0 ± 7.2 dB better audibility provided by the CI1 relative to HA which is a difference of 2 test step-sizes (Table 1B).

### Aural preference is revealed by both speech detection and perception

**Figure 7:**
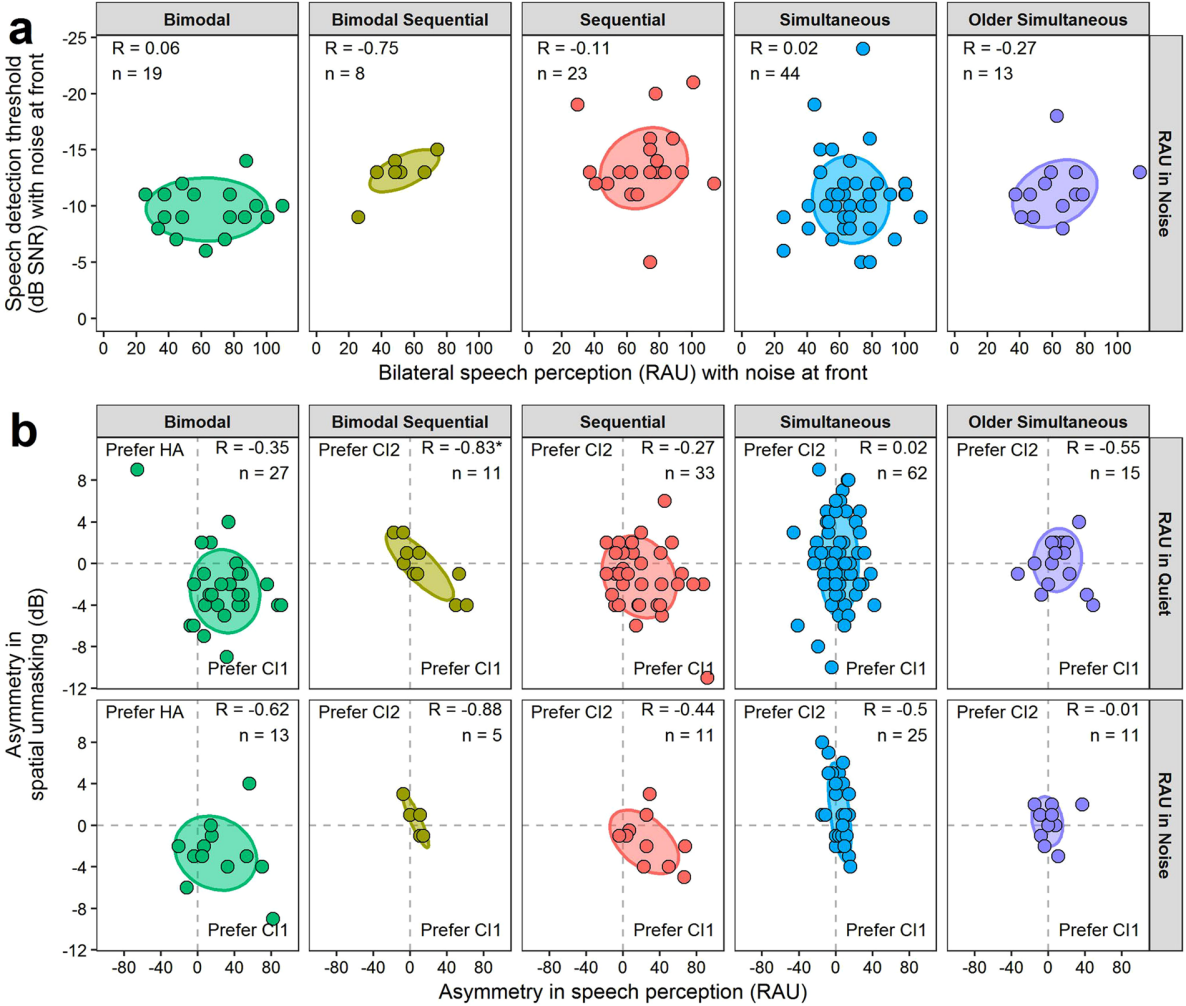
Relationship between speech detection and speech perception. (**a**) There was no relationship between speech detection thresholds in co-located noise (at front) and bilateral speech perception in co-located noise. (**b**) The relationship between asymmetry in spatial unmasking and asymmetry in speech perception tested in quiet and noise. For bimodal and sequential bilateral cochlear implant (CI) users, asymmetries in both measures favoured better performance with CI1 (bottom right quadrant). Pearson correlation coefficients (*R*) and number of children (*n*) are provided in each panel. *p < 0.05.

Of the 439 children with speech perception data, 148 (33.7%) also underwent spatial unmasking testing. Each test was completed within 4.7 ± 13.5 months of each other. Outcomes for each test are compared in Fig. 7. Bilateral speech perception scores in co-located noise at front did not correlate with speech detection thresholds in co-located noise at front (*p* > 0.05; Fig. 7a) and asymmetry in speech perception tested in quiet or noise did not correlate with asymmetry in spatial unmasking (*p* > 0.05, except *p* = 0.01 for bimodal sequential users for speech perception asymmetry in quiet). Yet, both measures revealed consistent preference for CI1 (Fig. 7b) in bimodal and sequential bilateral CI users. The ±1 SD (68% of children) distributions fall in the quadrant indicating a preference for CI1 in both measures; they had better speech perception scores when using CI1 and more benefit from spatially separating speech and noise when CI1 had the better signal-to-noise ratio (Fig. 7b). In contrast, the two simultaneous group distributions did not cluster in one quadrant, indicating no overall preference for one ear on either measure.

### Asymmetry increases with delayed or poor access to bilateral sound

Etiology did not predict speech perception asymmetry (ANOVA: *F*(4,419) = 0.7, *p* = 0.58) or spatial unmasking asymmetry (*F*(4,158) = 0.2, *p* = 0.94) (see Supplemental Fig. 4). Rather, asymmetry was predicted by the time- and hearing-based variables in the children’s diverse hearing histories. Correlations were first completed to identify associations between speech perception asymmetry and PCA components, as they captured different aspects of bilateral device users’ pre-implantation hearing. In quiet, speech perception asymmetry increased with lower PC2 scores (more unilateral deafness) (*R* = −0.19, *p* < 0.001, *n* = 350) and higher PC3 scores (any deafness) (*R* = 0.16, *p* < 0.001, *n* = 350). Effects of individual variables comprising significant PCA associations were then assessed, along with the following variables not included in the PCA: delay to bimodal/bilateral implantation and bilateral device use at time of testing. Considering all children irrespective of group, speech perception asymmetry increased with delay to bimodal/bilateral implantation (*R* = 0.32, *p* < 0.001, *n* = 431) and unaided hearing thresholds (worse residual hearing) in the HA/CI2 ear (*R* = 0.12, *p* = 0.011, *n* = 408).

**Figure 8:**
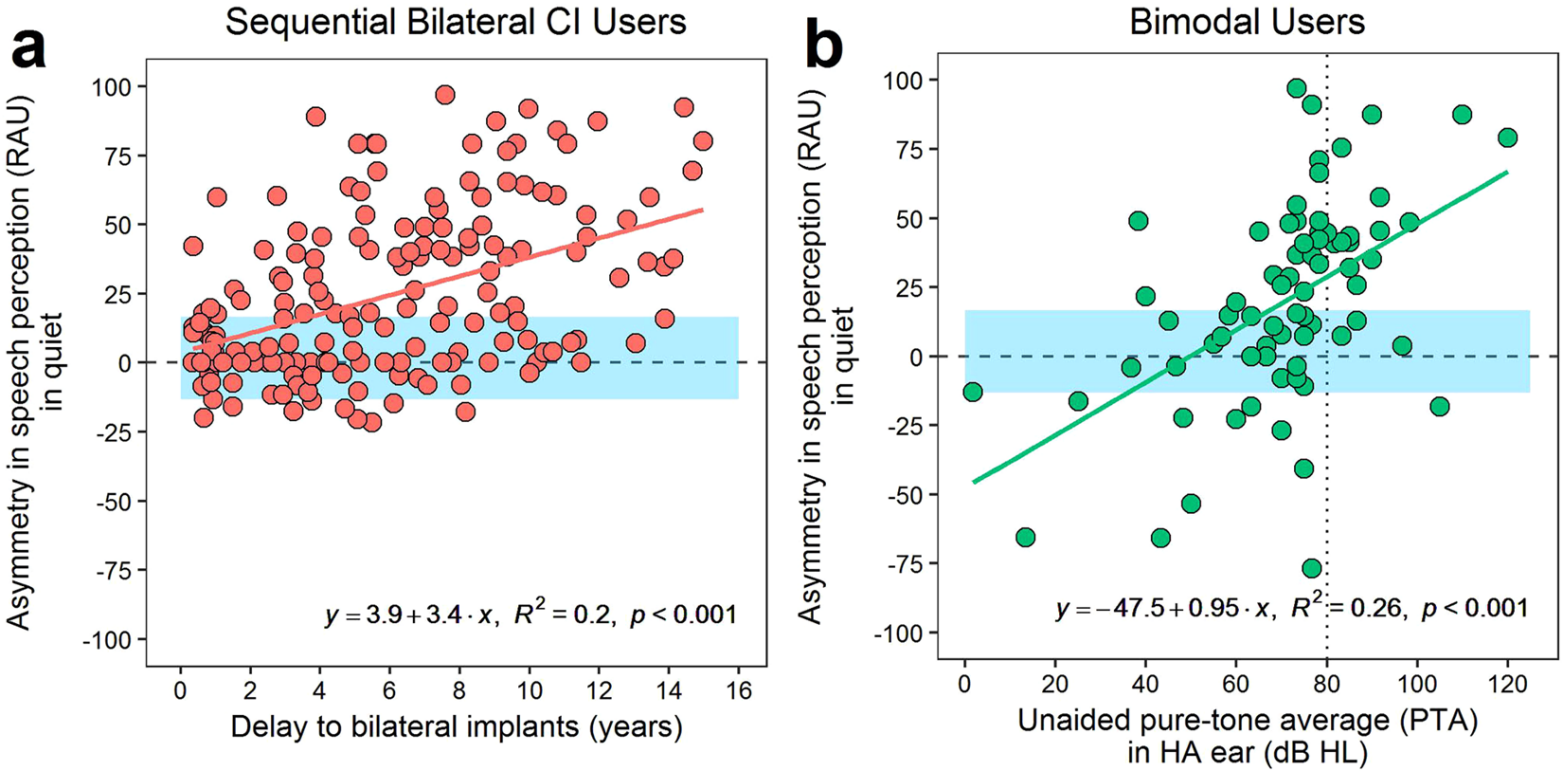
Changes in speech perception asymmetry in quiet. Asymmetry in speech perception tested in quiet increases as a function of (**a**) delay to bilateral implantation in sequential bilateral cochlear implant (CI) users, and (**b**) unaided pure-tone average (PTA) of hearing thresholds for 0.5, 1, and 2 kHz in the non-implanted ear with the hearing aid (HA) of bimodal users. The dotted vertical line in (**b**) indicates a severe-to-profound hearing loss (80 dB HL). Blue shaded regions indicate ±1 SD asymmetry of the simultaneous group for comparison. CI = cochlear implant; RAU = rationalized arcsine unit.

These relationships were particularly strong in sequential bilateral CI users and bimodal users respectively. Bivariate regressions in each group suggested that asymmetry increased by 3.9 RAU/year delay to bilateral implantation in sequential bilateral CI users (Fig. 8a; *F*(1,166) = 41.2, *p* < 0.001, *R*^2^_*adjusted*_ = 0.20), and increased by 9.5 RAU/10 dB worsening of hearing thresholds in the HA ear of bimodal users (Fig. 8b; *F*(1,71) = 25.6, *p* < 0.001, *R*^2^_*adjusted*_ = 0.26). This means that bimodal users had similar symmetry to simultaneous users (±1 SD = ±15 RAU) when they had mild to moderately-severe hearing loss in the non-implanted ear (35–65 dB HL unaided PTA). Otherwise, they showed aural preference for CI1 when the non-implanted ear had severe/profound loss (PTA > 70 dB HL) or aural preference for the HA ear when that ear had near-normal hearing (PTA < 35 dB HL). Sequential bilateral CI users developed an asymmetry >15 RAU when delay to bilateral implantation exceeded 3.5 years.

## Discussion

This study evaluated effectiveness, timing, and asymmetry of bilateral input in a large, diverse, and inclusive cohort of children who hear with bimodal devices or bilateral cochlear implants. Results demonstrated that: (1) speech recognition is better with bilateral than unilateral hearing; (2) bilateral hearing does not need to be restricted to one modality; and (3) asymmetric hearing creates functional aural preference for the better/first ear, limiting bilateral/spatial hearing. These findings are consistent with electrophysiological evidence of aural preference and support the recommendation to avoid this developmental change by providing access to sound with the most appropriate device in each ear as soon as possible.

Regardless of hearing history, most children recognized speech better (by 9.2 ± 14.0 RAU) when listening with two devices over one ear alone (Figs 4 and 5) even when this involved adding their poorer performing ear. This bilateral advantage is consistent with the 10–15% advantage reported in smaller cohorts of bilateral implant users with simultaneous^1^ or short (<2 year) inter-implant delays^1,41^, but is larger than the 0–10% reported for children with longer (>2 years) inter-implant delays^1,40,43^ or who use bimodal devices^46^. Including more children with limited auditory deprivation and better residual hearing likely contributed to the greater bilateral benefit exhibited by the present cohorts of sequential bilateral CI and bimodal users. It is also worthwhile noting that the bilateral advantage was further pronounced in noise (Fig. 4c), highlighting the utility of bilateral hearing in challenging listening situations.

Bilateral device users also adeptly detected speech when noise levels exceeded those of speech (Fig. 6a). Similar detection thresholds of −10 to −15 dB speech-to-noise ratio (SNR) were observed in peers with normal hearing^33,37^ or bilateral CIs^33,34,37^. Measures of speech detection allowed testing of children with wide ranges of age, native language, and developmental ability but detection is an easier task than speech recognition. This explains the lower SNR levels in this study compared to the −9 to +7 dB SNR speech recognition thresholds reported for children with normal hearing^55,56^ or CIs^39,44,45,57^. The ability to detect and understand speech in noisy conditions improves with age^33,55,56^ as the auditory system matures. Changes continue into adulthood^37^ with the largest changes occurring over the first 5 years^55,56^. In the present cohort, detection thresholds did not change with age when accounting for group, perhaps because most children were older than 5 years and had early access to sound (Table 1; Supplemental Table 4).

The advantage of bilateral hearing existed for both bimodal and bilateral CI hearing. This suggests that the auditory system can integrate, at least to some extent, two very different auditory signals to facilitate listening in quiet and noisy situations. Indeed, even limited input from a HA can work with a CI to help bimodal users perceive speech and develop language^46,58–62^. By having better residual hearing and access to sound than previous bimodal users^46^, the present cohort of bimodal users exhibited improvement in speech perception and detection on par with their peers using two CIs (Figs 4b and 6a). Like bilateral CIs, bimodal devices adequately stimulate the bilateral auditory pathways to promote symmetric brainstem development^13^ and typical hemispheric representation of sound^27,28^ when provided with limited delay to children with sufficient residual hearing. For the deaf ear, a CI outperforms a HA: the 30.5 ± 23.8 RAU bilateral benefit over listening with a HA alone (Fig. 4b) supports expanding implantation criteria to allow bimodal hearing in children with some residual hearing. This, along with asymmetric speech perception (Fig. 8b), also suggests that bimodal users with poor residual hearing in their non-implanted ears may fare better with bilateral implants. Remaining residual low-frequency hearing has delayed implantation so that temporal fine structure for music and emotion perception, not available through CI use^63^, can be maintained^46,64,65^. However, children using bimodal devices with little residual hearing (PTA > 80 dB HL) do not perceive music or emotion better than their peers with bilateral implants^66–68^. The greatest bilateral advantage may be derived through the most appropriate bilateral devices for the hearing loss in each ear, regardless of modality.

Although there was clear benefit of listening bilaterally rather than unilaterally with bilateral CIs and bimodal hearing, most children developed asymmetric function which limited this advantage (Fig. 5). This was particularly evident in children who experienced asymmetric hearing or unilateral deprivation (Fig. 8) and consequently developed aural preference for the better/first hearing ear (Figs 3, 6 and 7). Moreover, this preference persisted despite several years of bilateral device use (Table 1A). These lasting deleterious effects of asymmetric hearing in development are consistent with neurophysiological findings including persistent asymmetries in brainstem development^69^ and increased cortical representation from the better than poorer hearing ear^21,22,28^.

For most children, asymmetric speech perception reflected poorer performance in the second or non-implanted ear relative to the first implanted ear. Whereas scores consistently exceeded 50 RAU for the first implanted ear, scores when listening with the other ear varied from 0 to 100 RAU (Fig. 3). The resulting range of asymmetries was similar to previous reports (~20–40% asymmetry in sequentially implanted children^1,41,43^ and 0–5%^1^ asymmetry or 0.9–1.1 CI2/CI1 ratio^42^ in simultaneously implanted children). Also consistent with previous studies^1,42^, delay to bilateral input contributed to asymmetric speech perception (Figs 3, 5 and 8). Asymmetric speech perception leaves children with limited bilateral hearing (Fig. 5) during sensitive periods in development. Although consistent daily implant use can improve speech perception in both ears^2,70^, asymmetry did not correlate with bilateral device use in this study. Rather, delaying sufficient access to sound during development created asymmetric hearing with prolonged consequences of deteriorated speech perception and limited benefit of bilateral input.

Aural preference also affected spatial hearing. All groups showed ~3–4 dB spatial unmasking which was poorer than the normal range of 5–10 dB^1,35,37,55,56^, but within the 2–4 dB range reported for sequential bilateral CI and bimodal users^33,35–37,44,45^. More importantly, simultaneous groups took similar advantage of spatial unmasking when noise moved to either side (Fig. 6)^1^ whereas children who had asymmetric speech perception detected speech 1.2–2.5 dB better when noise moved towards the poorer/second ear than the better/first ear (Figs 6 and 7). This asymmetric use of spatial separation in bimodal and sequential groups falls within the lower range of previously reported 1.8–4 dB asymmetries favouring a better signal to the first implanted ear^33,36,37,45^. The degree of asymmetry measured may have implications for everyday function. Even an improvement of 1–2 dB in spatial unmasking can reduce self-rated difficulties in situations containing background noise and reverberation^71^, and small increases in SNR can improve speech intelligibility^72^. Poorer (reduced) SNR on one side signals a potential for reduced speech perception in the condition when the “preferred” or better ear is masked by noise. Although asymmetries in spatial hearing can decrease after 3 years of bilateral input as the second ear’s performance improves^45^, the present cohort used their bilateral devices for 3–6 years, suggesting that these aural asymmetries are long-lasting.

Bilateral implants provided similar audibility from each device for all children (Table 1B) but only sequentially implanted children exhibited asymmetric spatial unmasking. This suggests that the aural preference for their first implanted ear is not simply a function of audibility in each ear but includes ignoring/neglecting information from the worse ear. By contrast, bimodal users had poorer hearing thresholds when using the HA than when using the CI and thus experienced both periods of asymmetric hearing and asymmetric audibility in each ear (Table 1B). The asymmetric access to sound with the two different types of hearing devices may exacerbate the asymmetry in spatial hearing, potentially explaining why bimodal users exhibited the largest spatial unmasking asymmetry.

This study is the first to directly compare asymmetries measured by two different measures of speech perception. Children exhibiting asymmetric speech perception also showed asymmetric spatial hearing that favoured the first implanted ear (Fig. 7b). This agreement between the two behavioural measures suggests an underlying functional/behavioural aural preference. Notably, electrophysiological measures similarly assert an underlying premise for aural preference that emphasizes the importance of minimizing delay to bilateral hearing in bimodal and bilateral implant users. Extensive reorganization of auditory pathways occurs with asymmetric hearing during development, leading to an over-representation of the better ear^13,21,23,24,27–31,69^. Brainstem asymmetries^69^ and cortical aural preference^21,28^ favouring the first hearing/implanted ear occur within 2–3 years of asymmetric input. Years of subsequent bilateral input cannot simply reverse aural preference^21,28^, unless bilateral input is provided quickly and at early ages^21,27^. Similarly, stronger aural preference occurs with reduced age of unilateral hearing in deaf white kittens^23,24^ and with temporary asymmetric hearing in ferrets and rats^29–31^. Persistently asymmetric speech perception occurs when bilateral input is delayed for ~3.5–4 years as shown in Fig. 8a and in previous studies^1,40–43,45^. The slightly staggered timelines suggest that underlying neurophysiological changes develop rapidly and take ~1–2 years longer to translate into measurable and consistent functional changes. This could reflect increasing complexity of speech perception, which requires temporal and spectral processing, relative to responses in temporal (auditory) cortices evoked by brief broadband click/pulse stimuli^28^.

By including children with short or no delays to bilateral implantation, this study is one of few to directly provide behavioural evidence that corroborates electrophysiological findings about the most appropriate timing of bilateral input. Simultaneous bilateral cochlear implantation can be performed safely in young children who are bilaterally deaf ^73^ without increased risk for complications or cumulative costs^74–76^. Moreover, simultaneous bilateral implant users develop symmetric representations of each ear in the auditory cortex^20,21^, symmetric speech perception (Fig. 3)^1^, equal bilateral advantage over either ear (Fig. 4), and equal spatial unmasking when noise moves to either ear (Fig. 6)^33^. Yet, as with any clinical population, individual variability exists and some asymmetries could occur even when early bilateral input is provided simultaneously (Fig. 7a). Contributing factors may include ear differences in: hearing loss progression, cochlear shape, insertion and position of the electrode array, neural integrity, stimulation consistency due to external device malfunctions, or pitch mismatches that may affect binaural fusion into one auditory image. Despite these potential sources of variability, the present data assert that providing early simultaneous bilateral input will give children the best chance of developing speech perception (reaching open-set word identification at a younger age shown in Fig. 2) and avoiding aural preference.

In summary, the present study contributes behavioural corroboration to electrophysiological evidence of an “aural preference syndrome” that develops with both unilateral and asymmetric bilateral hearing during childhood. To avoid these deteriorations in hearing, our findings show that bilateral devices appropriate for the hearing loss in each ear should be provided early and without delay. Although children were not randomly assigned to treatment groups, the evidence presented here is consistent with several other studies of functional outcomes and underlying neurophysiological changes. A randomized control trial is unlikely to yield findings that would markedly change the clinical recommendations suggested here. Promoting early bilateral auditory development as soon as possible maximizes the opportunity for children to develop symmetric speech perception and spatial hearing; skills that are not only important for listening and navigating in complex environments^8^ but also for academic and social success^10,77–79^.

## Methods

### Participants

All methods were performed in accordance with the study protocol #1000002954 approved by the Hospital for Sick Children’s Research Ethics Board. Parental consent was obtained for all participants. All available speech detection and recognition outcomes as well as demographic information between 2001-02-25 and 2017-06-20 (16.3 years) were collected from 461 children with bilateral devices: 80 (17.4%) children who used one cochlear implant (CI) and had normal hearing or used a hearing aid (HA) in the contralateral ear (“Bimodal”); 18 (3.9%) bimodal users who received a second CI (“Bimodal Sequential”); 170 (36.9%) children who received two CIs in sequential surgeries but did not wear a HA during the delay (“Sequential”); 193 children who received two CIs in the same surgery, 154 (33.4%) before age 4 years (“Simultaneous”) and 39 (8.5%) after age 4 years (“Older Simultaneous”). Most implants were from Cochlear Ltd, except for 5 children who received an Advanced Bionics array in their first implanted ear. Most children received a peri-modiolar CI24RE internal electrode array (56.0% of CI1; 73.5% of CI2); the type of array was unknown for 11 (2.4%) CI1 and 4 (1.1%) CI2 (details are provided in Supplemental Fig. 5). Group demographic details regarding first implanted ear, gender and whether the hearing loss was asymmetric are described in Table 1A. Asymmetric hearing loss was defined as: 1) hearing loss better than profound in one ear; 2) asymmetry ≥10 dB HL at 3 adjacent frequencies and/or pure-tone-average (PTA) asymmetry ≥15 dB HL.

### Etiology of deafness differs by group

**Figure 9:**
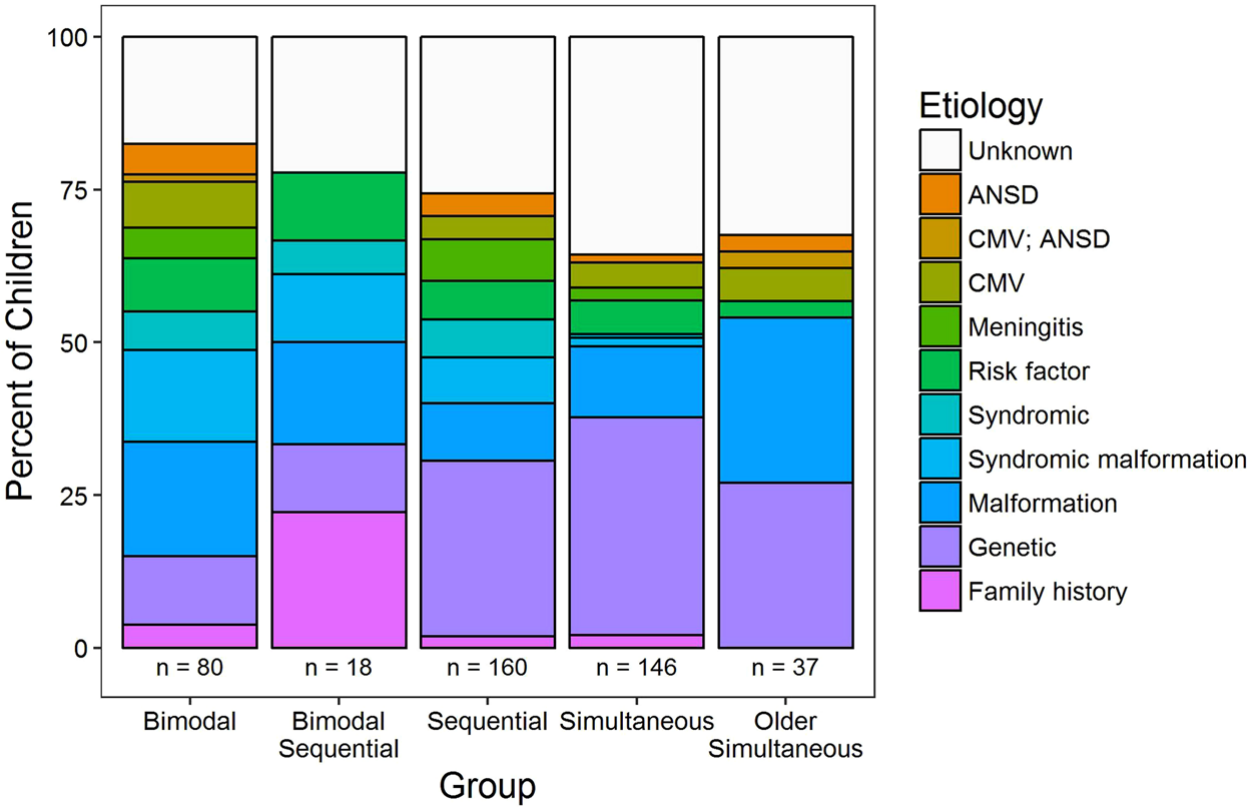
Etiology of deafness by group. Distribution of known (coloured) and unknown (gray) etiologies of deafness in each group. Knowing family history (pink), genetic (purple) or radiological findings (blues) accounted for at least 50% of etiologies of deafness for each group. Numbers of children (*n*) in each group are provided.

The distribution of known and unknown etiologies of deafness is shown for each group in Fig. 9. Genetic, radiological and medical history information was available for 441/461 children (95.7%). With this information, etiology of hearing loss was identified in at least 50% (51.0–66.7%) of children in each group. Etiology was unknown in 17.5–36.0% of children in each group. Etiological distributions partitioned bimodal device from young bilateral CI users: consistent with their congenital bilateral deafness (Table 1), children with bilateral CIs had a higher rate of genetic (e.g., GJB2, MTRNR1, DFNB, MITF, MYO7A/15A, LOXHD1 mutations; *Χ*^2^(4) = 17.1, FDR-adjusted p = 0.005) or family history (*Χ*^2^(3) = 22.5 FDR-adjusted *p* = 0.001) etiology of deafness, whereas bimodal users and older simultaneous bilateral CI users had a higher rate of malformations which are often associated with progressive and/or asymmetric hearing loss (e.g., enlarged vestibular aqueduct (EVA), incomplete partition type II (IP-II/Mondini), Cock’s dysplasia (common cavity, CC) or cochlear hypoplasia connected with Pendred, CHARGE, Branchio Oto Renal (BOR), X-linked deafness with stapes gusher (Phelp’s) and Klippel Feil syndromes; *Χ*^2^(3) = 15.7, FDR-adjusted *p* = 0.005).

### Speech perception in quiet and co-located noise

#### Tests

Speech perception was evaluated in both quiet and co-located + 10 dB SNR noise using words presented at 60 dB SPL through a loudspeaker at 0° azimuth in a sound-treated audiometric booth. Speech perception tests were chosen based on language and developmental stage of the child. The following tests were used at the earliest and latest (or only) test dates, ordered by difficulty: Early Speech Perception test (earliest:latest ESP pattern/words; *n* = 7:2/23:2); Digit Identification (Digits; *n* = 3:5); Word Identification by Picture Identification (WIPI; *n* = 20:9); Glendonald Auditory Screening Procedure (GASP words; *n* = 94:22); Multisyllabic Lexical Neighbourhood Test (MLNT words; *n* = 92:67); and Phonemic Balanced Kindergarten test (PBK words; *n* = 201:333). Children responded by either pointing to a picture best representing the heard word from a group of pictures (closed-set: ESP, WIPI) or repeating the heard word (closed-set: Digits; open-set: GASP, MLNT, PBK). Because number of words varied across tests (12–25 words), percent correct scores were transformed to rationalized arsine units (RAU) and then corrected for guessing on closed-set tests^80,81^.

#### Administration

All tests but the PBK were administered with monitored live voice and used speech-weighted noise. PBK tests (predominant test in available data) were administered with recorded words and the associated multi-talker noise in most cases; sometimes speech-weighted noise was used due to technical difficulties, and for some children live voice was used because of their language, developmental stage or attention abilities. Quiet and noise PBK scores from a subset of children (*n* = 181, 62.8%) were analyzed and confirmed that test administration differences did not affect speech perception in quiet (CI1, CI2/HA, bilateral), calculated asymmetry, or bilateral advantage across groups (see Supplementary material, Supplemental Table 2, and Fig. 6). Significant effects of noise and presentation (recorded versus monitored live voice) were found for absolute speech perception scores in noise and thus were included as covariates in group analyses for this condition.

#### Conditions

In quiet, 439 children listened to words presented from the front while wearing one device at a time (unilaterally), and 374 (85.2%) of these children also listened to words while wearing both devices at the same time (bilaterally). Scores were also obtained in noise for CI1 and for the bilateral condition for 288 (65.6%) of the children. Of these, 94 (32.6%) children also had unilateral scores for both ears in noise. Repeated tests were available in quiet for both ears in 312/439 (71.1%) children and for bilateral scores in 309/374 (82.6%) children, as well as in noise for the better ear (CI1) and bilateral conditions in 272/288 (94.4%) children. Data were available for CI2 in noise for 89/94 (94.7%) children. A detailed breakdown of numbers by group, ears tested and condition is provided in Supplemental Table 3A.

To evaluate changes across repeated tests completed by the same child, scores were converted to a Pediatric Ranked Order Speech Perception (PROSPER) score^82^, which hierarchizes score (<50%, ≥50%) and type of test into one score that could be followed over time. This accounts for a possible change in score by virtue of moving from a simpler to more challenging test. If two tests were available on a given test date, the more difficult test for that child was chosen. The number of available test dates ranged from 2–11, with a median of 3 tests per child (Supplemental Table 3B). Mean ± SD ages at the earliest and latest test dates were 7.3 ± 4.1 and 10.6 ± 4.1 years old respectively, for a difference of 3.3 ± 2.3 years (see Supplemental Table 3C for a breakdown by group). To evaluate speech performance in all children, scores from the latest or only available date were used, giving the greatest proportion of similar tests administered (PBK; 333/439 (75.8%)) and greatest chance for any asymmetry between ears to resolve.

### Spatial unmasking (speech detection in co-located and separated noise)

Children wore both devices (bilateral) during testing. Speech detection thresholds (SDT) were measured as described previously^33,71^. Briefly, recorded speech was presented from a loudspeaker at 0° azimuth in the presence of speech-weighted noise presented at a level of 60 dB SPL at 0° and ±90° azimuth. The speech stimulus consisted of a male talker repeatedly saying ‘bup-bup-bup’. Level of the speech adaptively changed in 2 dB increments. Spatial unmasking described the benefit obtained when noise was spatially moved away from the speech and was calculated as: SDT with noise at 0°– SDT with noise at ±90°. Data were available for 171 children, 38 (22.2%) of whom performed the test more than once. Most of these children were tested on 2 occasions (Supplemental Table 4). When multiple sessions were available, the latest date was chosen for cross-sectional analyses of all children. Children were 8.4 ± 4.1 and 9.6 ± 4.3 years old at the earliest and latest tests respectively, with a difference of 1.3 ± 0.5 years.

### Statistical analyses

Principle component analysis (PCA) was completed on demographic variables in the hearing history rather than step-wise regression because many variables were correlated and would introduce collinearity into a multiple linear regression model. This also allowed for variables that described a similar aspect of hearing history to be combined into one component that guided correlation analyses with outcome measures.

ANOVA was used to assess group differences in a number of measures: demographic variables provided in Table 1; principle component values; asymmetry in speech perception; asymmetry in spatial unmasking; and asymmetry in speech perception across etiology. Repeated measures ANOVA was used to assess main effects and interactions for group differences (between-subject factor) where conditions were repeated (within-subject factor): bilateral speech perception scores in quiet versus in noise; bilateral advantage for speech perception over using CI1 or CI2/HA alone; bilateral advantage in quiet versus in noise; speech detection thresholds with noise directed to the front, CI1 or CI2/HA; and spatial unmasking with noise moved from the front to CI1 versus CI2/HA. Greenhouse-Geisser corrections for lack of sphericity were used when indicated by a significant Mauchly test of sphericity. Tukey’s honest significant difference (HSD) post-hoc testing was completed for significant effects in the ANOVA and repeated measures ANOVA to account for family-wise error in multiple comparisons.

The asymmetry in speech perception and in spatial unmasking was assessed in order to further highlight the capabilities and challenges of bilateral device users. Asymmetry between CI1 and CI2/HA was further assessed using Lin’s concordance correlation coefficient^83,84^, which assesses the extent to which points conform to the line of best fit (correlated) and how far that line is from the unity line (perfect agreement). This analysis quantified asymmetry in each child while accounting for the absolute accuracy of scores or unmasking in each ear, which is lost when only difference measures (i.e., the asymmetry) are provided. This complexity and richness in information is important to retain while evaluating the wide variability in outcomes in diverse groups of bilateral device users. Furthermore, Lin’s coefficient accounts for differences/deviations from agreement (i.e., symmetry) (whereas Pearson correlations do not). This way, the complexity and variability of the individual data across children who had poor scores and good scores could be highlighted alongside asymmetry. An ANOVA analysis assessed group differences in speech perception asymmetry, bilateral advantage and spatial unmasking asymmetry. The significance of these difference measures were analyzed compared to zero using independent *t*-tests with false-discovery rate (FDR) corrections to *p*-values for multiple comparisons^85^.

Pearson correlations were used to assess the following relationships between the following outcome measures: bilateral advantage versus absolute asymmetry in speech perception; bilateral speech detection thresholds versus bilateral speech perception in noise coming from the front; and asymmetry in spatial unmasking versus asymmetry in speech perception. Correlations were also completed to identify relationships between asymmetry in speech perception and the PCA components of hearing history. These correlations guided which demographic variables were considered for regression analyses in order to predict changes in speech perception asymmetry.

Linear mixed effects regression models^86^ were used to assess changes speech perception asymmetry over time, as well as the progression from easier to harder tests of speech perception (PROSPER score) with bilateral device experience. These mixed regressions were used instead of simple linear regression in order to account for repeated measures per child and to allow for individual variation in the relationship over time. To do this, random effects of both intercept and slope for each child (1 + years|subject) were added to the regression model. A likelihood ratio test was used to determine significance of predictors in the regression. The equation of the full linear mixed effects model is given below:

$${\rm{score}} \sim {\rm{years}}+{\rm{group}}+{\rm{years}}:{\rm{group}}+({\rm{1}}+\mathrm{years}|\mathrm{subject})$$

Visual inspection of residual plots for each analysis did not reveal any obvious deviations from homoscedasticity or normality.

## Electronic supplementary material

Supplementary Material

## Data Availability

The data supporting the findings of this study are available from the corresponding author upon reasonable request.
