# Supplementary material for: Limiting asymmetric hearing improves benefits of bilateral hearing in children using cochlear implants
Source: Sci Rep. 2018 Sep 4;8:13201. doi: 10.1038/s41598-018-31546-8 (PMC6123397; doi:10.1038/s41598-018-31546-8)
Supplement: Supplementary file 1 — Supplementary Material [file 41598_2018_31546_MOESM1_ESM.pdf]

Limiting asymmetric hearing improves benefits of bilateral hearing in children using cochlear implants – Supplemental Information

Melissa Jane Polonenko*[1,2], Blake Croll Papsin[1,3,4], Karen Ann Gordon[1,2,3,4]

* Melissa Polonenko, melissa.polonenko@mail.utoronto.ca, Archie's Cochlear Implant Laboratory, The Hospital for Sick Children, Room 6D08, Toronto, ON, M5G 1X8, Canada

Blake Papsin, blake.papsin@sickkids.ca, Otolaryngology, The Hospital for Sick Children, Toronto, ON, M5G 1X8, Canada

Karen Gordon, karen.gordon@utoronto.ca, Archie's Cochlear Implant Laboratory, The Hospital for Sick Children, Room 6D08, Toronto, ON, M5G 1X8, Canada

Supplemental Data

Supplemental Table 1. Factor loading matrix for principal component analysis (PCA) of demographic variables of hearing history

Component description:	Asymmetric hearing experience	Unilateral deafness	Any deafness	Bilateral deafness	Asymmetry with poor hearing	Residual hearing	Hearing asymmetry	Asymmetry versus unilateral deaf	Early intervention
**Variable:**	**PC1**	**PC2**	**PC3**	**PC4**	**PC5**	**PC6**	**PC7**	**PC8**	**PC9**
Age received CI1	**-0.73**	**0.40**	-0.33	**0.33**	-0.03	0.19	-0.14	0.00	**-0.19**
Time: pre-CI acoustic hearing	**-0.87**	0.26	-0.10	0.13	**-0.21**	0.19	-0.16	0.01	**0.23**
Time: asymmetric hearing	**-0.79**	**-0.42**	-0.28	-0.03	-0.05	-0.18	0.12	**-0.28**	0.00
Time: unilateral deafness	**-0.71**	**-0.50**	**-0.37**	-0.05	-0.06	-0.15	0.12	**0.26**	-0.01
Time: bilateral deafness	0.51	0.20	**-0.47**	**0.42**	**0.51**	-0.14	0.10	0.00	**0.08**
Pre-CI unaided PTA: CI1 ear	0.49	**-0.70**	-0.12	0.19	0.03	0.10	**-0.46**	-0.02	0.00
Pre-CI unaided PTA: CI2/HA ear	**0.68**	-0.23	**-0.31**	0.11	**-0.31**	**0.43**	**0.31**	-0.02	0.01
Asymmetric hearing: pre-CI	-0.59	-0.23	0.15	**-0.25**	**0.61**	**0.38**	0.06	0.00	0.00
Asymmetric hearing: post-CI	-0.34	-0.29	**0.59**	**0.65**	-0.01	-0.02	0.16	0.02	0.00
**PCA summary:**									
Eigenvalue	**3.83**	**1.36**	**1.03**	0.83	0.78	0.49	0.42	0.15	0.10
Proportion of variance	0.43	0.15	0.11	0.09	0.09	0.05	0.05	0.02	0.01
Cumulative proportion	0.43	0.58	0.69	0.78	0.87	0.93	0.97	0.99	1.00

Factor loadings are bolded and shaded for variables which contributed proportionally more to the principal component than what would be expected if variables contributed equally (i.e., > 100% / 9 variables = 11.1%). PTA = pure-tone-average of .5, 1, 2 kHz. CI = cochlear implant; HA = hearing aid; PC = principal component; PCA = principal component analysis

Longitudinal changes in asymmetry in speech perception

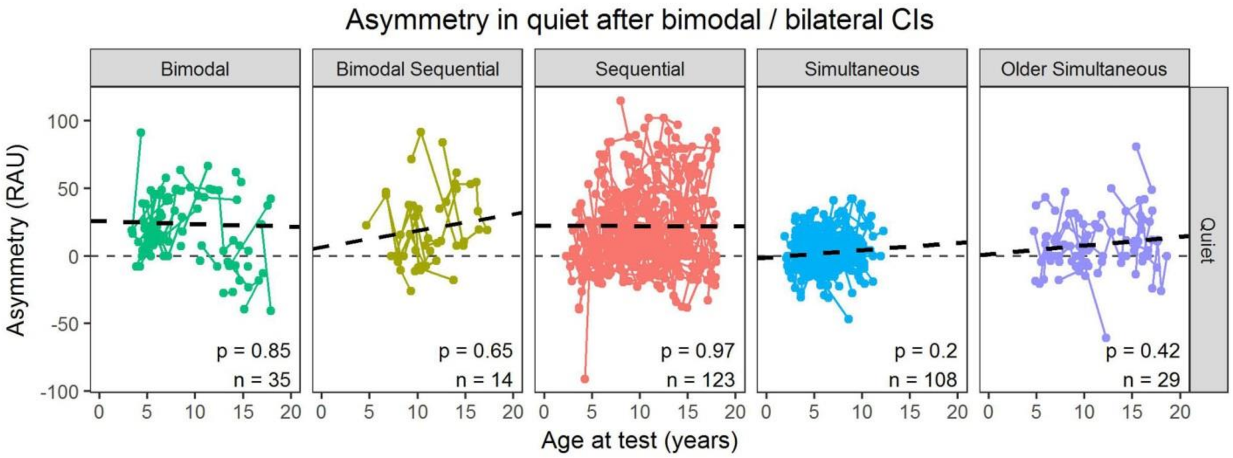

Supplemental Figure 1. Longitudinal changes in asymmetry in speech perception with age. The difference in speech perception (asymmetry) in quiet between the first implanted ear and second implanted or hearing aid ear did not change with age at test. Coloured lines join repeated results for each child; dashed black lines indicate non-significant (*p*<0.05) group changes in asymmetry with age based on linear mixed-effects regression. The number of children (*n*) is provided for each group.

Bilateral advantage of speech perception

Bilateral advantage over listening with one ear in noise is shown in Supplemental Figure 2a, and over listening with CI2/HA alone in both quiet and noise is shown in Supplemental Figure 2b. Bilateral advantage over listening with only the HA/CI2 was greater than over listening with CI1 alone (*F*(1,87)=7.8, *p*=0.007) and there was no interaction with group (*F*(4,87)=2.1, *p*=0.09), reflecting overall asymmetry between unilateral conditions. There was a main effect of group (*F*(4,87)=3.5, *p*=0.011), whereby average bilateral benefit for simultaneous users was 12.2±4.2 RAU less than sequential users (*z*=-2.9, *p*=0.026) and 12.6±3.9 RAU less than bimodal users (*z*=-3.2, *p*=0.011). The bilateral advantage over listening with HA/CI2 was not different in quiet versus noise (*F*(1,95)=0.0, *p*=0.82), but there remained a significant effect of group (*F*(4,95)=3.4, *p*=0.011). Average bilateral advantage over CI2 (left ear) alone for simultaneous users was 13.5±4.6 RAU less than sequential users (*z*=-3.0, *p*=0.025) and 14.1±4.4 RAU less than the bilateral advantage over the HA alone of bimodal users (*z*=-3.2, *p*=0.012).

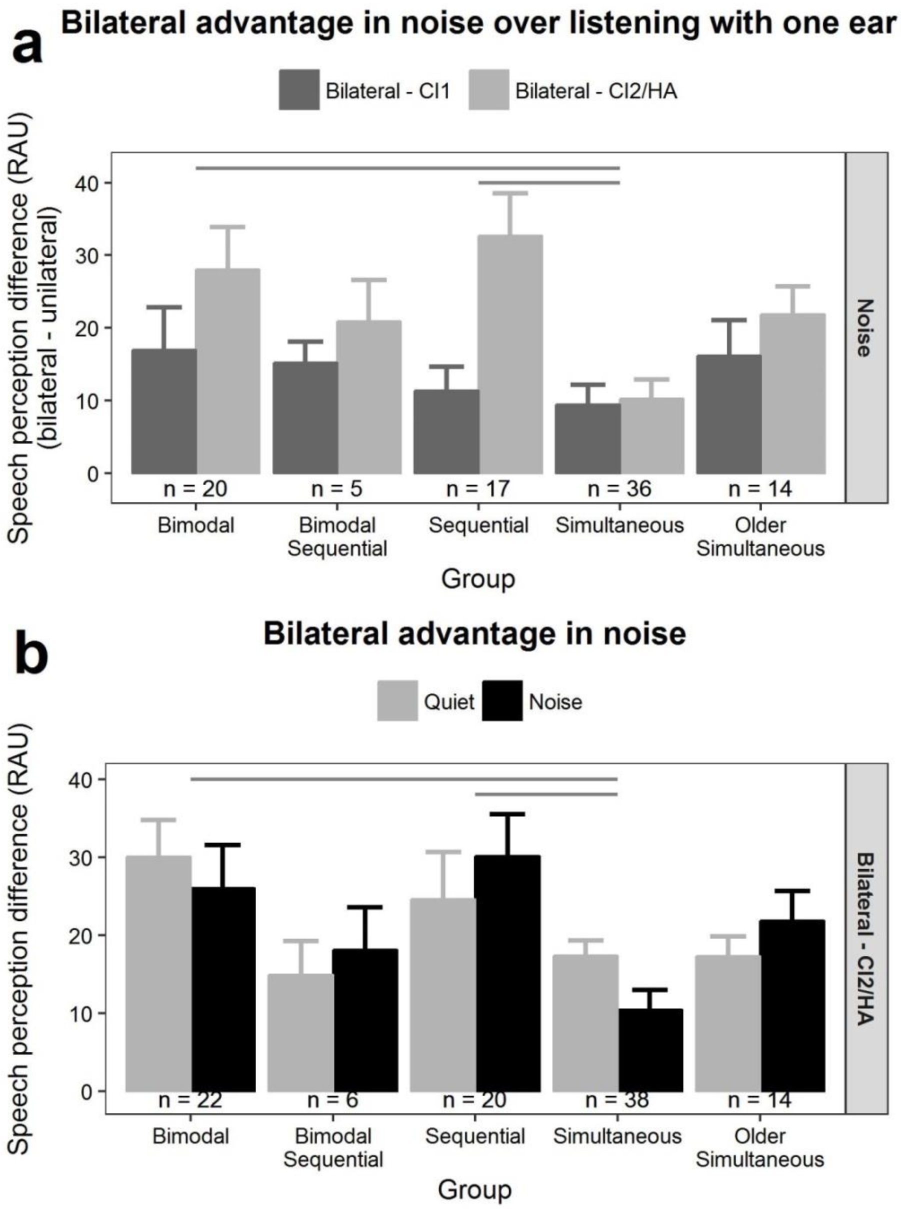

Supplemental Figure 2. Bilateral advantage for speech perception in noise. (a) The difference in speech perception in rationalized arcsine units (RAU) while testing in noise for the bilateral condition over listening with each ear alone. Bilateral advantage over listening with only the HA/CI2 was greater than over listening with CI1 alone. Average bilateral benefit for simultaneous users was less than sequential and bimodal users. (b) Bilateral advantage over listening with HA/CI2 alone in both quiet and noise. Bilateral advantage differed by group but not by whether there was noise present. Average bilateral advantage over CI2 alone for simultaneous users was less than sequential users and less than the bilateral advantage over the HA alone of bimodal users.

Supplemental Figure 3 shows the advantage of bilateral input to speech perception over listening with only CI1. As the asymmetry between unilateral scores increasingly favoured CI1 (positive asymmetry values), the advantage of adding HA/CI2 decreased (all FDR-corrected $p<0.05$). For sequential and some older simultaneous users who had large asymmetries favouring CI1, adding CI2 decreased speech perception accuracy. On the other hand, for bimodal and some simultaneous users who had asymmetries favouring the HA/CI2 (left CI for simultaneous group), the bilateral advantage was greatest because the better perceiving ear was given access to sound. This negative correlation between asymmetry and bilateral advantage also occurs when considering bilateral advantage over the best performing ear: advantage to providing bilateral input decreased as absolute asymmetry increased ($p<0.05$ for all groups; Supplemental Figure 3).

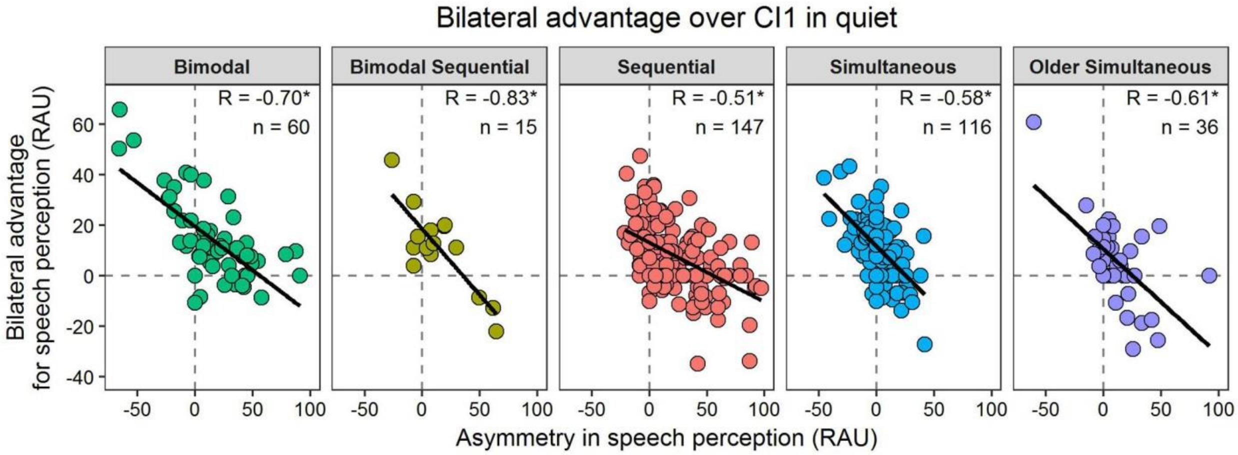

Supplemental Figure 3. Best advantage to bilateral input with symmetric hearing. The advantage to speech perception in quiet was calculated as the difference between scores with bilateral input and the first implanted ear. This bilateral advantage decreased as the absolute value of the asymmetry in speech perception between ears increased. Asterisks indicate significant correlation after FDR corrections for multiple comparisons ($p<0.05$).

Etiology alone does not predict speech perception asymmetry in quiet

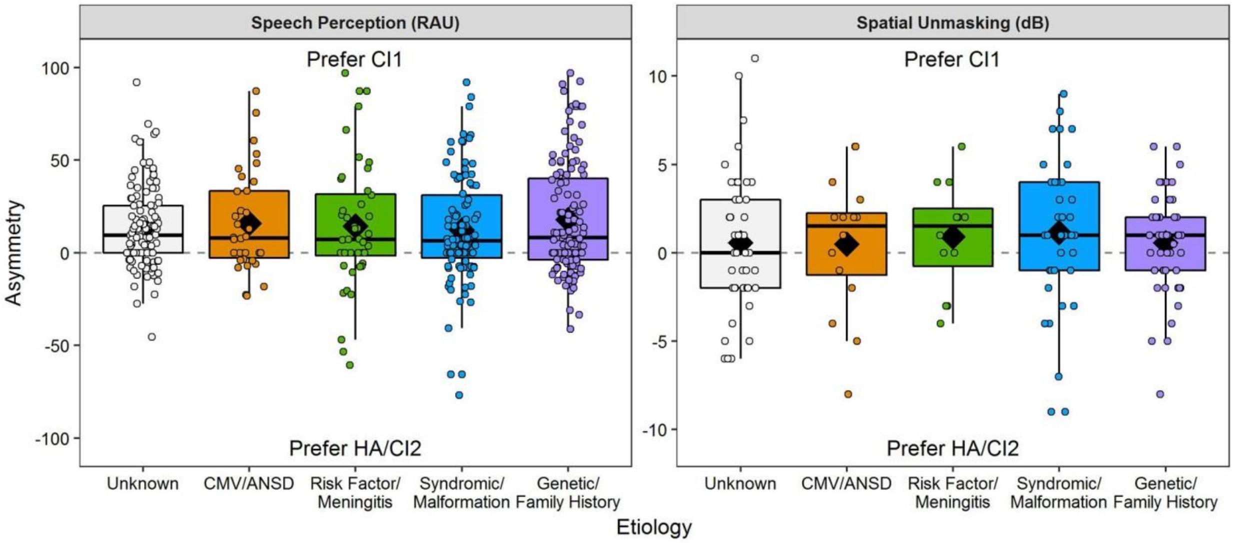

Supplemental Figure 4. Asymmetry in speech perception and spatial unmasking by etiology. Asymmetries in speech perception in quiet and spatial unmasking were similar across etiology of deafness. Means are shown by the black diamonds. Values for spatial unmasking were multiplied by -1 (i.e., flipped) so that all positive values indicate a preference for the first implanted ear (CI1). CI = cochlear implant; HA = hearing aid; CMV=congenital cytomegalovirus; ANSD=auditory neuropathy spectrum disorder; RAU=rationalized arcsine unit

Demographic information

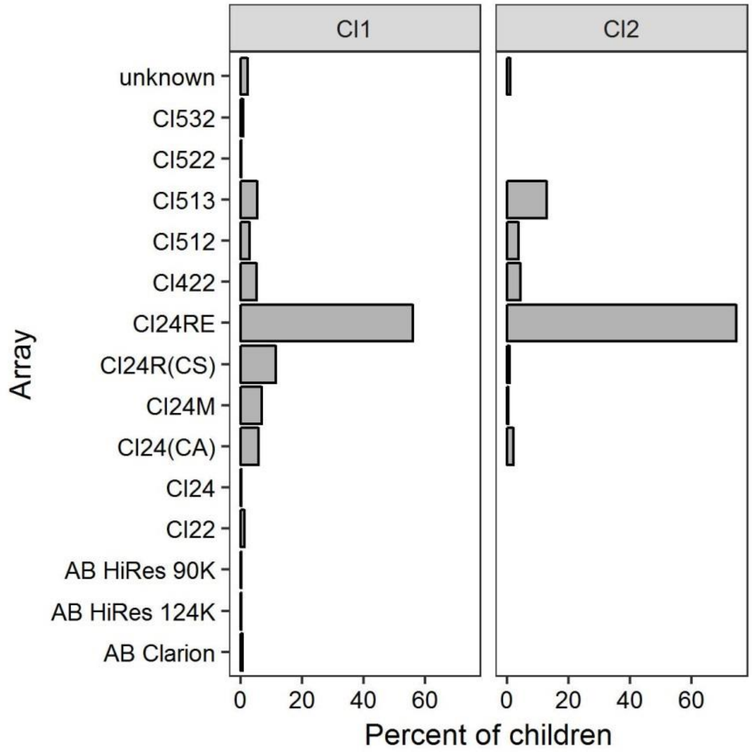

Supplemental Figure 5. Distribution of internal arrays for each implanted ear. Percentage of children with each type of internal array implanted in each ear (CI1:CI2 n = 461:375). Most implants were from Cochlear Ltd, except for 5 children who received an Advanced Bionics (AB) array in their first implanted ear. Most children received a CI24RE in both ears. The type of array was unknown for 11 (2.4%) CI1 and 4 (1.1%) CI2.

Test delivery affects speech perception scores in noise but not measures of asymmetry or bilateral advantage

To determine the effect of test delivery on speech perception measures, repeated measures ANOVA was completed on a subset of children (181/288 = 62.8%) who were tested in both quiet and noise with the most common and challenging test (PBK), using ear (bilateral, CI1, CI2/HA) or difference (asymmetry, bilateral advantage over CI1, bilateral advantage over CI2/HA) as a within-subject factor, method (monitored-live voice, recorded) and group as between-subject factors, and age as a covariate. Recorded speech stimuli were used in 123/181 (68.0%) children tested in quiet and 100/141 (70.9%) children tested in noise. Speech-

weighted noise was used with monitored-live voice delivery and when multi-talker noise could not be used with recorded stimuli due to technical difficulties. However, most of the time multi-talker noise was used with recorded PBK delivery. The number of children by group, condition, ear(s) tested and delivery method are provided in Supplemental Table 2.

Supplemental Table 2. Number of children tested in each group and condition

**Condition**	**Group**	**Ear tested**	**Stimulus Delivery**		**Total**
			**Live Voice**	**Recorded**	
Quiet	Bimodal	CI1, Bilateral	14	17	31
		CI2/HA	14	17	31
	Bimodal Sequential	CI1, Bilateral	4	7	11
		CI2/HA	4	7	11
	Sequential	CI1, Bilateral	14	42	56
		CI2/HA	14	42	56
	Simultaneous	CI1, Bilateral	20	41	61
		CI2/HA	20	41	61
	Older Simultaneous	CI1, Bilateral	6	16	22
		CI2/HA	6	16	22
Noise	Bimodal	CI1, Bilateral	10	13	23
		CI2/HA	5	9	14
	Bimodal Sequential	CI1, Bilateral	2	7	9
		CI2/HA	1	4	5
	Sequential	CI1, Bilateral	9	37	46
		CI2/HA	3	11	14
	Simultaneous	CI1, Bilateral	15	30	45
		CI2/HA	12	23	35
	Older Simultaneous	CI1, Bilateral	5	13	18
		CI2/HA	3	10	13

Speech perception scores and difference measures are shown in Supplemental Figure 6. In quiet, method of stimulus delivery did not significantly affect speech perception scores ($F(1,170)=3.0$, $p=0.08$) or difference measures ($F(1,170)=0.0$, $p=0.88$), and there were no interactions between method and ear tested (scores: $F(2,340)=1.5$, $p=0.23$; differences: $F(2,340)=2.6$, $p=0.10$) or group (scores: $F(4,170)=0.4$, $p=0.84$; differences: $F(4,170)=0.1$, $p=0.97$). Although speech perception scores in noise were better for live voice than for recorded stimuli ($F(1,70)=10.7$, $p=0.002$), this effect was consistent for each ear ($F(2,140)=0.8$, $p=0.43$) and group ($F(4,70)=0.0$, $p=0.99$). Consequently, stimulus delivery did not affect asymmetry or bilateral advantage (difference scores) in noise (main effect: $F(1,70)=0.2$, $p=0.62$, method*group: $F(4,70)=0.3$, $p=0.84$; method*difference score: $F(2,140)=1.3$, $p=0.26$).

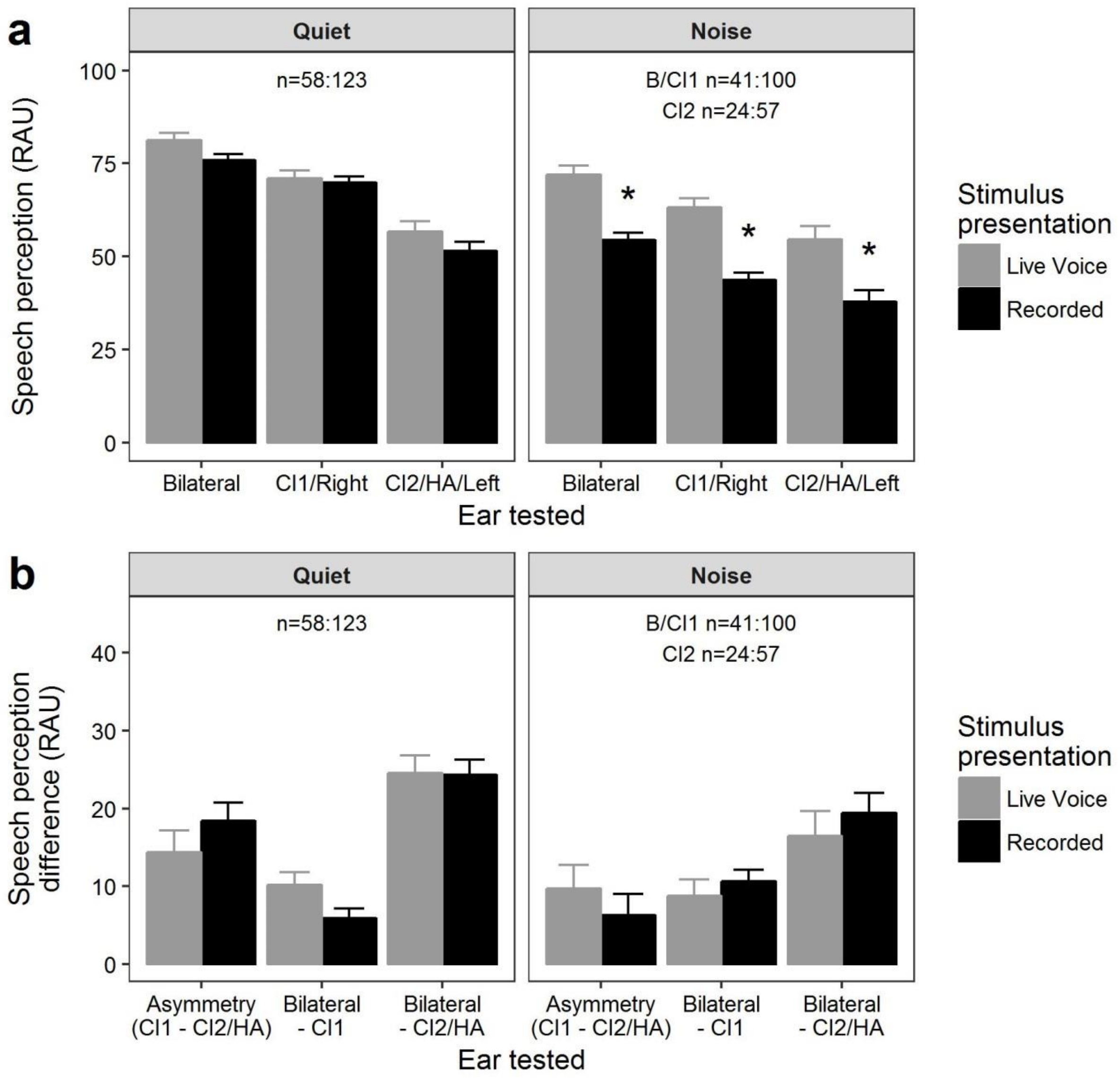

Supplemental Figure 6. Effect of stimulus delivery method on speech perception outcomes. Mean ± SE speech recognition scores for (a) each ear(s) tested and (b) the resulting differences between ears are shown for children who were tested in quiet and noise with speech delivered either by monitored live voice (gray) or with recorded stimuli (black). For the noise condition, speech-weighted noise was used with monitored-live voice presentation and sometimes with recorded stimuli when technical difficulties precluded the typical multi-talker babble noise. Most children were tested with recorded stimuli; numbers of children tested are provided for live voice:recorded stimulus presentation. Delivery method only affected the absolute score in noise; there was no effect on measures of asymmetry or bilateral advantage. * $p<0.05$; B = bilateral; CI = cochlear implant; HA = hearing aid

Numbers of children with available data

Supplemental Table 3. Number of children and test dates with available speech perception scores

**A. Number of children**								
**Group**	**Cross-sectional data: *n*(%)**				**Longitudinal data: *n*(%)**			
	**Quiet**		**Noise**		**Quiet**		**Noise**	
	**CI1 & CI2/HA**	**Bilateral, CI1 & CI2/HA**	**CI1 & CI2/HA**	**Bilateral & CI1**	**CI1 & CI2/HA**	**Bilateral, CI1 & CI2/HA**	**CI1 & CI2/HA**	**Bilateral & CI1**
Bimodal	73	61 (83.6)	20 (27.4)	39 (53.4)	38 (52.1)	28 (45.9)	0 (0.0)	12 (30.8)
Bimodal Sequential	17	14 (82.4)	5 (29.4)	10 (58.8)	14 (82.4)	13 (92.9)	3 (60.0)	8 (80.0)
Sequential	168	147 (87.5)	18 (10.7)	121 (72.6)	123 (73.2)	118 (80.3)	2 (11.1)	108 (88.5)
Simultaneous	142	116 (81.7)	37 (26.1)	90 (62.7)	108 (76.1)	93 (80.2)	15 (40.5)	73 (82.0)
Older Simultaneous	39	36 (92.3)	14 (35.9)	28 (71.8)	29 (74.4)	27 (75.0)	2 (14.3)	21 (75.0)
Total	439	374 (85.2)	94 (21.4)	288 (65.6)	307 (69.9)	279 (74.6)	22 (23.4)	223 (77.4)

**B. Number of tests in longitudinal data**					**C. Age at earliest and latest tests**			
**Group**	**Longitudinal data: Range (median)**				**Longitudinal data: age at test**			
	**Quiet**		**Noise**		**Mean ± SD (years)**		**Range (years)**	
	**CI1 & CI2/HA**	**Bilateral, CI1 & CI2/HA**	**CI1 & CI2/HA**	**Bilateral & CI1**	**Earliest test**	**Latest test**	**Earliest test**	**Latest test**
Bimodal	2-4 (2)	2-4 (2)	n/a	2-3 (2)	8.2±4.1	9.9±4.2	3.4-16.9	4.4-17.9
Bimodal Sequential	2-6 (3)	2-6 (3)	2-3 (2)	2-5 (2.5)	10.2±3.2	13.3±2.4	4.7-16.1	9.3-17.3
Sequential	2-11 (3)	2-10 (3)	2-3 (2.5)	2-10 (3)	8.8±4.1	12.7±4.0	2.3-17.5	4.2-18.0
Simultaneous	2-8 (3)	2-7 (3)	2-3 (2)	2-7 (3)	4.1±1.2	7.5±1.9	1.9-7.3	2.9-12.2
Older Simultaneous	2-10 (3)	2-10 (3)	2-3 (2.5)	2-10 (3)	10.3±4.1	12.3±4.1	4.7-17.1	5.9-18.6
Total	2-11 (3)	2-10 (3)	2-3 (2)	2-10 (3)	7.3±4.1	10.6±4.1	1.9-17.5	2.9-18.6

Supplemental Table 4. Number of children and tests with available spatial unmasking scores

Group	N(%) of children		No. of tests	Mean ± SD age (years)		Age range (years)	
	Cross-sectional	Longitudinal	Range (median)	Earliest test	Latest test	Earliest test	Latest test
Bimodal	35	7 (20.0)	2-3 (2)	6.5±3.8	7.4±3.8	4.1-14.8	4.6-15.5
Bimodal Sequential	12	6 (50.0)	2-2 (2)	12.2±1.3	13.5±1.6	10.3-13.9	11.3-15.6
Sequential	35	8 (22.9)	2-3 (2)	13.2±1.6	14.6±1.9	11.1-15.5	11.8-17.1
Simultaneous	74	16 (21.6)	2-3 (2)	5.6±2.5	6.9±2.6	1.5-9.2	2.1-10.8
Older Simultaneous	15	1 (6.7)	2-2 (2)	5.9	6.9	5.9	6.9
Total	171	38 (22.2)	2-3 (2)	8.4±4.1	9.6±4.3	1.5-15.5	2.1-17.1
